# Nitrate as a potential prebiotic for the oral microbiome

**DOI:** 10.1038/s41598-020-69931-x

**Published:** 2020-07-30

**Authors:** B. T. Rosier, E. Buetas, E. M. Moya-Gonzalvez, A. Artacho, Alex Mira

**Affiliations:** Department of Health and Genomics, Center for Advanced Research in Public Health, FISABIO Foundation, Avenida de Catalunya 21, 46020 Valencia, Spain

**Keywords:** Microbial ecology, Microbiome, DNA sequencing, Next-generation sequencing, Biofilms

## Abstract

The salivary glands actively concentrate plasma nitrate, leading to high salivary nitrate concentrations (5–8 mM) after a nitrate-rich vegetable meal. Nitrate is an ecological factor that can induce rapid changes in structure and function of polymicrobial communities, but the effects on the oral microbiota have not been clarified. To test this, saliva of 12 healthy donors was collected to grow in vitro biofilms with and without 6.5 mM nitrate. Samples were taken at 5 h (most nitrate reduced) and 9 h (all nitrate reduced) of biofilm formation for ammonium, lactate and pH measurements, as well as 16S rRNA gene Illumina sequencing. Nitrate did not affect biofilm growth significantly, but reduced lactate production, while increasing the observed ammonium production and pH (all p < 0.01). Significantly higher levels of the oral health-associated nitrate-reducing genera *Neisseria* (3.1 ×) and *Rothia* (2.9 ×) were detected in the nitrate condition already after 5 h (both p < 0.01), while several caries-associated genera (*Streptococcus*, *Veillonella* and *Oribacterium*) and halitosis- and periodontitis-associated genera (*Porphyromonas*, *Fusobacterium*, *Leptotrichia*, *Prevotella*, and *Alloprevotella*) were significantly reduced (p < 0.05 at 5 h and/or 9 h). In conclusion, the addition of nitrate to oral communities led to rapid modulation of microbiome composition and activity that could be beneficial for the host (i.e., increasing eubiosis or decreasing dysbiosis). Nitrate should thus be investigated as a potential prebiotic for oral health.

## Introduction

The salivary glands actively concentrate plasma nitrate into the saliva, which leads to fasting levels of salivary nitrate in the 100–500 μM range (i.e., approximately 10 times higher than in plasma) (reviewed in REFS^1,2^). After a nitrate containing meal, this mechanism causes a further increase of salivary nitrate concentrations up to 5–8 mM^2^, which remain elevated many hours due to the long half-life circulation of nitrate^3^. The food groups that naturally contain most nitrate are vegetables and fruits, both generally associated with health benefits, lower disease prevalence and longevity^4,5^. Certain oral bacteria reduce nitrate (NO_3_^−^) mostly to nitrite (NO_2_^−^), but also further to more reactive nitrogen intermediates such as nitric oxide (NO) in a process called denitrification^6^.

Importantly, a significant amount of the produced nitrite is swallowed and taken up into the blood circulation of the host, where it is converted into nitric oxide—a signaling molecule involved in cardiovascular and metabolic regulation^4,7^. This is referred to as the nitrate-nitrite-nitric oxide pathway and provides an oral microbiome-dependent way of obtaining bioactive nitric oxide in addition to the classical NO synthases of the host^1^. Described systemic effects from nitrate supplementation include lowering of blood pressure, improved endothelial function, increased exercise performance, reversal of metabolic syndrome and anti-diabetic effects^4^. The importance of nitrate-reducing oral bacteria is reflected by the observation that blood pressure acutely increases in fasting individuals after using chlorhexidine mouthwash resulting from the loss of oral nitrate reduction^7^. Additionally, chlorhexidine mouthwash interferes with post-exercise hypotension^8^ and over-the-counter mouthwash usage correlated with pre-diabetes and diabetes development^9^.

Importantly, inside the oral cavity, salivary nitrate, nitrite and the nitrate reducing capacity of the oral microbiome have been proposed to be beneficial to prevent caries^10–12^. Additionally, two weeks of nitrate-rich lettuce juice consumption improved gingival health in a recent clinical study^13^. Finally, in other recent clinical intervention studies^14,15^, 1–4 weeks of high doses of nitrate in the form of concentrated beetroot juice increased the number of health-associated nitrate-reducing bacteria in saliva^16^. It is therefore important to determine experimentally if nitrate can be considered a dietary component associated with oral health.

While different studies have shed light on the molecular mechanisms involved in the systemic effects of nitrate reduction by the oral microbiota^1,4,7,17^, mechanisms leading to the apparent health-associated roles inside the oral cavity remain largely hypothetical. To unravel these mechanisms, the effect of nitrate on oral communities must be studied. Oral microorganism in saliva form biofilms on tooth surfaces (i.e., dental plaque) and the tongue (i.e., tongue coating)^16,18^. These biofilms are involved in the development of the most common oral diseases, including caries, periodontitis and halitosis.

It must be born in mind that nitric oxide has antimicrobial properties and planktonic cultures of certain oral species associated to periodontitis have shown to be sensitive to this highly oxidative compound^19^. It would therefore be crucial to determine if the reduction of nitrate affects the composition of oral communities by inhibiting the growth of disease-associated species, while increasing health-associated nitrate-reducing species. Furthermore, the capacity of oral bacteria to reduce nitrite to the alkali molecule ammonia, and to use lactic acid as a carbon source during denitrification could both prevent acidification, which is responsible for caries development^6,12^.

In relation to this, in a pioneering study, Koopman et al. (2016)^20^ applied 5 mM nitrate pulses of 6 min to 1–4 week old oral microcosms (i.e., complex, large in vitro oral biofilms) from two individuals, and each of them responded differently to nitrate. However, an effect on pH buffering was not detected and the number of participants was too low to conclude how the biofilm composition may be affected by nitrate.

In our current study, the effect of a single dose of nitrate on freshly sampled oral communities was tested in vitro. In short, saliva of 12 healthy donors was incubated in nutrient-rich medium with or without the physiologically relevant concentration of 6.5 mM nitrate in an impedance-based system (xCELLigence) that monitors real-time biofilm growth^21,22^. Samples were taken after 5 h, when most nitrate was reduced and some nitrite was produced, and at 9 h, when all nitrate and most nitrite were metabolized. Supernatant samples were taken for the measurements of nitrate, nitrite, ammonium, lactate and pH. The remaining biofilms were collected for protein and DNA quantification, as well as Illumina sequencing of the 16S rRNA gene. By this experimental protocol, we aimed to study the short-term effect of a single dose of nitrate on pH, oral biofilm growth and bacterial composition.

## Materials and methods

### Unstimulated saliva sampling

For this study, adults who reported to be systemically healthy were recruited as saliva donors at the FISABIO Institute (Valencia, Spain). Individuals were excluded if frank cavitation was detected at the moment of sampling (following the criteria of Rosier et al., 2017^23^, which was assessed visually by an experienced dentist, or any history of periodontitis (following the criteria of Camelo-Castillo et al., 2015^24^, as well as if they had used antibiotics or regularly used oral antiseptics in the previous month.

Twelve healthy donors were instructed to have a normal breakfast and abstain from oral hygiene before saliva collection in the morning. Five mL of unstimulated saliva were collected at least one hour after breakfast by drooling^25^ in a sterile tube in a quiet room. The saliva was used for in vitro growth and biofilm quantification. The procedure was repeated another time for biofilm sequencing and supernatant analysis.

To determine the effect of nitrate on acidification due to glucose fermentation, 9 healthy donors were asked to donate saliva under fasting conditions (abstaining from breakfast and oral hygiene) to avoid the presence of dietary-derived salivary nitrate.

The fresh unstimulated saliva was always directly used in the experiments or kept at 4 °C for < 1 h before usage. All donors gave informed consent prior to sample collection and the protocol was approved on 2016/05/23 by the Ethical Committee of DGSP-FISABIO (Valencian Health Authority) with the reference BIO2015-68711-R2. This study was carried out according to the relevant guidelines and regulations of the Declaration of Helsinki.

### In vitro oral biofilm growth and impedance-based quantification

Unstimulated saliva of twelve donors was grown in ‘E-Plate 96’ 96-well plates in the xCELLigence system (ACEA Biosciences, San Diego, California, USA). Each E-Plate is coated with a golden layer at the bottom of the wells that is connected to microelectrodes, allowing the measurement of biofilm growth in real-time^21,22,26,27^. The impedance formed by biofilm adherence has been shown to be proportional to biofilm mass, which is provided by a corresponding Cell Index and expressed in arbitrary units^28^. Previous sequencing of biofilms grown under these circumstances show that bacterial composition is representative of different oral niches, depending on the sample type used for inoculation^22^.

BHI medium (Biolife, Deerfield, Illinois, USA) with an additional 0.05 mg/L haemin, 0.005 mg/L menadione and 0.2 mM vitamin K (all Sigma-Aldrich, St. Louis, Missouri, USA) was prepared of which 100 µL was added to each well for background impedance measurements. Additional 25 µL of 65 mM nitrate (NaNO_3_, Sigma-Aldrich) in water or just water was added to each well of the nitrate or control condition, respectively. Then, 125 µL freshly collected saliva was added, leading to a final concentration of 6.5 mM nitrate (within the 5–8 mM physiological range of saliva after a nitrate containing meal) in the nitrate condition. The E-Plate 96 was placed in the xCELLigence system inside an incubator at 37 °C. Every 10 min, a Cell Index measurement was taken. All experiments were performed without agitation and anaerobic conditions were favored by sealing the wells with adhesive aluminum foil (VWR, Radnor, Pennsylvania, USA), which previously allowed the growth of strictly anaerobic bacteria^22^. All conditions (control 5 h, control 9 h, nitrate 5 h, nitrate 9 h) were grown in duplicate.

For 0 h measurements, 1:1 medium and saliva mixtures were used. After 5 h and 9 h of growth, the supernatant was sampled and stored at − 20 °C until pH, nitrate, nitrite, ammonium and lactate measurements were performed in duplicate. A PBS washing step was then performed to remove unattached cells and, after this, the remaining cells were removed with a pipette and resuspended in 100 µL PBS. Biofilm duplicates were resuspended together in 200 µL PBS for storage at − 20 °C until DNA isolation for sequencing.

The washing step removed part of the (slightly attached) bacteria that had accumulated after 5 h or 9 h at the bottom of the well. Therefore, for protein and DNA quantification, the PBS washing step was not performed to quantify the entire microbial community that affected the physiological measurements in our in vitro system (e.g., pH, lactate and ammonium). After removing supernatant, the biofilms were resuspended in 75 µL PBS. It was observed that nitrate affects the impedance of the xCELLigence system and this effect depended on the saliva of the donor. Therefore, controls with microorganism-free filtered saliva were used to normalize the cell-index measurements. For this, the saliva was first filtered with a 5 µm filter and then with 0.1 µm filter.

### Incubating saliva with nitrate and glucose

The unstimulated saliva of nine donors collected under fasting conditions was used to test the effect of different concentrations of nitrate on a pH drop caused by 0.2% of glucose after 5 h of incubation. For each donor, 187 µL of saliva and 22 µL of glucose (2% diluted in water) was added per well of a standard 96-well plate. Then, 11 µL of water without or with different concentrations of nitrate was added, leading to final concentrations of 0 mM, 0.5 mM, 1 mM, 1.5 mM, 2.5 mM, 3.5 mM, 4.5 mM, 5.5 mM, 6.5 mM ,7.5 mM and 8.5 mM of nitrate. The plate was sealed with adhesive aluminum foil and incubated during 5 h at 37 °C. After incubation, the samples were stored at − 20 °C until pH measurements.

### Nitrate, nitrite, ammonium, lactate and pH measurements

For the nitrate, nitrite, ammonium, lactate and pH measurements, the RQflex 10 Reflectoquant (Merck Millipore, Burlington, Massachusetts, USA) reflectometer was used. This method is based on the intensity of reflected light by two reactive pads on test strips that change in color intensity based on the concentration of a specific substance^29^.

The test strips (Reflectoquant, Merck Millipore) for pH had a range from pH 4–9, the strips for nitrate a range of 3–90 mg/L, the strips for nitrite a range of 0.5–25 mg/L, the strips for ammonium a range of 5–20 mg/L, and the strips for lactate a range of 3–60 mg/L. Accuracy of all reflectometer methods was confirmed by the use of standard solutions (nitrate, nitrite, ammonium, all Merck Millipore; lactate, BioVision, Milpitas, California, USA) with known concentrations of the different compounds.

A method was used based on Hemke et al. (2009) and Ferrer et al. (2020)^30,31^. In short, undiluted supernatant was used for pH measurements and, for the rest of the measurements, 10 × or higher dilutions were made to obtain a concentration within the detection threshold of the test strips. Then, 15 µL of (diluted) supernatant was added to each of the two reactive patches on a strip, and excess liquid was removed by tipping the side of the strip on a tissue.

Before nitrate measurements, the diluted supernatants in which 0.5 mg/L or more nitrite was detected were treated with amidosulfuric acid (Sigma-Aldrich) based on the manufacturer's instructions. For this, 35 µL of diluted supernatant was mixed with 1.5 µL amidosulfuric acid solution (10%).

For ammonium measurements, aliquots were made of 50 µL diluted supernatant to which 10 µL of reagent 1 (ammonium 5–20 mg/L test strip kit, Reflectoquant, Merck Millipore) was added first and resuspended well. Then, 15 µL of a freshly made mixture of reagent 2 dissolved in 1.25 mL water was added and resuspended. This solution was then directly added to the strips and incubated according to the manufacturer's instructions.

### Biofilms protein and DNA quantification

Biofilms grown for 5 h and 9 h were resuspended in 75 µL PBS of which 30 µL was used for protein quantification and the rest for DNA isolation. For protein quantification, the Bradford protein assay was applied, which is based on the colour change of the Coomassie Brilliant Blue dye (G-250) when bound to proteins. Duplicates of 15 µL of resuspended pellet were added to different wells of a standard 96-well plate. Then, 240 µL of Bradford Reagent (Sigma-Aldrich), containing G-250, was added and, after 5 min of incubation in the dark, the absorbance was measured with an Infinite F200 plate reader (TECAN, Männedorf, Switzerland) at 600 nm. Protein concentrations were determined using a calibration curve with known concentrations of BSA (range 0–1.5 mg/mL, Sigma-Aldrich) on each plate. For DNA quantification, DNA was extracted as described in the next section. Then, the DNA concentration was measured using the Qubit 1 × dsDNA HS Assay Kit and a Qubit 3 Fluorometer (both Thermo Scientific, Waltham, Massachusetts, USA), according to manufacturer’s instructions.

### Biofilm composition determined by 16 rRNA sequencing

#### DNA extraction for sequencing

For DNA extraction, the biofilm duplicates (or salivary pellets from 250 µL saliva for inoculum sequencing) were resuspended in 100 µL PBS and disaggregated 30 s in a sonicator bath (model VCI-50, Raypa, Barcelona, Spain) at low ultrasound intensity. After this, DNA was isolated by MagNA Pure LC 2.0 Instrument (Roche Diagnostics, Risch-Rotkreuz, Switzerland), using the MagNA Pure LC DNA Isolation Kit III for Bacteria and Fungi (Roche Diagnostics) following the manufacturer’s instructions with an additional enzymatic lysis step: to a bacterial pellet in 100 µL PBS, 130 µL lysis buffer and 2.5 µL of enzyme mix, containing 20 mg/mL lysozyme (Thermomixer comfort, Eppendorf, Hamburg, Germany), 5 mg/L lysostaphin (Sigma-Aldrich) and 0.625 mg/mL mutanolysin (Sigma-Aldrich), were added and incubated for 60 min at 37 °C. DNA was resuspended in 100 µL elution buffer and frozen at − 20 °C until further analysis. To determine the amount of DNA for sequencing, the Quant-iT PicoGreen dsDNA Assay Kit and a Qubit 3 Fluorometer (both Thermo Scientific) were used, according to manufacturer’s instructions.

#### 16 rRNA sequencing

A pre-amplification step of the V1–V5 regions of the 16S rRNA gene was performed, following Dzidic et al. (2018)^32^. An Illumina amplicon library was then performed following the 16S rRNA gene Metagenomic Sequencing Library Preparation Illumina protocol (Part #15044223 Rev. A), targeting the 16S rRNA gene V3 and V4 regions, resulting in a single amplicon of 460 bp. Amplicons were sequenced on a MiSeq Sequencer according to manufacturer’s instructions (Illumina, San Diego, California, USA) using the 2 × 300 bp paired-ends protocol.

#### Taxonomic classification

The sequences were analyzed according to Boix-Amorós et al. (2016)^33^. In short, the reads were quality-filtered and end-trimmed in 10 bp windows with Prinseq. The PCR chimeras were removed with UCHIME according to Edgar et al. (2011)^34^. Given that taxonomic assignment accuracy decreases dramatically in reads shorter than 200 bp^35^, single reads were discarded and only joined reads were used to be taxonomically assigned at the genus level with the classifier of the Ribosomal Database Project^36^, using a confidence interval of 80%. Operational Taxonomic Unit (OTU) selection was performed using VSEARCH^37^ at a 97% of sequence identity. Given that taxonomic accuracy gets reduced at species level, especially in some genera with a highly similar 16S rRNA gene among species, only sequences > 400 bp were used for species-level classification^38^. We aligned each OTU centroid using BLAST at 97% of identity and 100% query coverage and retrieved only those species that agreed with the previous classification of the centroid at genus level provided by RDP classifier^39^.

### Statistical analysis

We used overall R programming language for statistical computing^40^ to perform downstream analyses. Only those taxa with an abundance of > 0.001% in more than three samples in at least one condition were selected for the analyses. For multivariant analysis, an Adonis test (Permutational Multivariate Analysis of Variance Using Distance Matrices), provided by the Vegan library of R^41^, was used to compare groups. To visualize groups and their differences in a two-dimensional map, we computed constrained principal components via constrained correspondence analysis (CCA) which is also part of Vegan library^41^. For univariate analyses, paired non-parametric Wilcoxon tests (i.e., “wilcox.test” function of stats library of R^40^ were performed to test the differences in genera and all other parameters between groups. Correlations within and between genera and other parameters were determined with Spearman's rho, along with associated p-value using the “cor.test” function of the stats library of R^40^. Finally, to see if the observed taxonomic changes were supported by a standard compositional data analysis (CODA) technique, an ANCOM-II analysis was performed according to Kaul et al. (2017)^42^, which also controls the false discovery rate at a desired level of significance. For all taxonomic comparisons and correlations, only adjusted p-values were used.

## Results

### Effect of nitrate on biofilm growth

**Figure 1:**
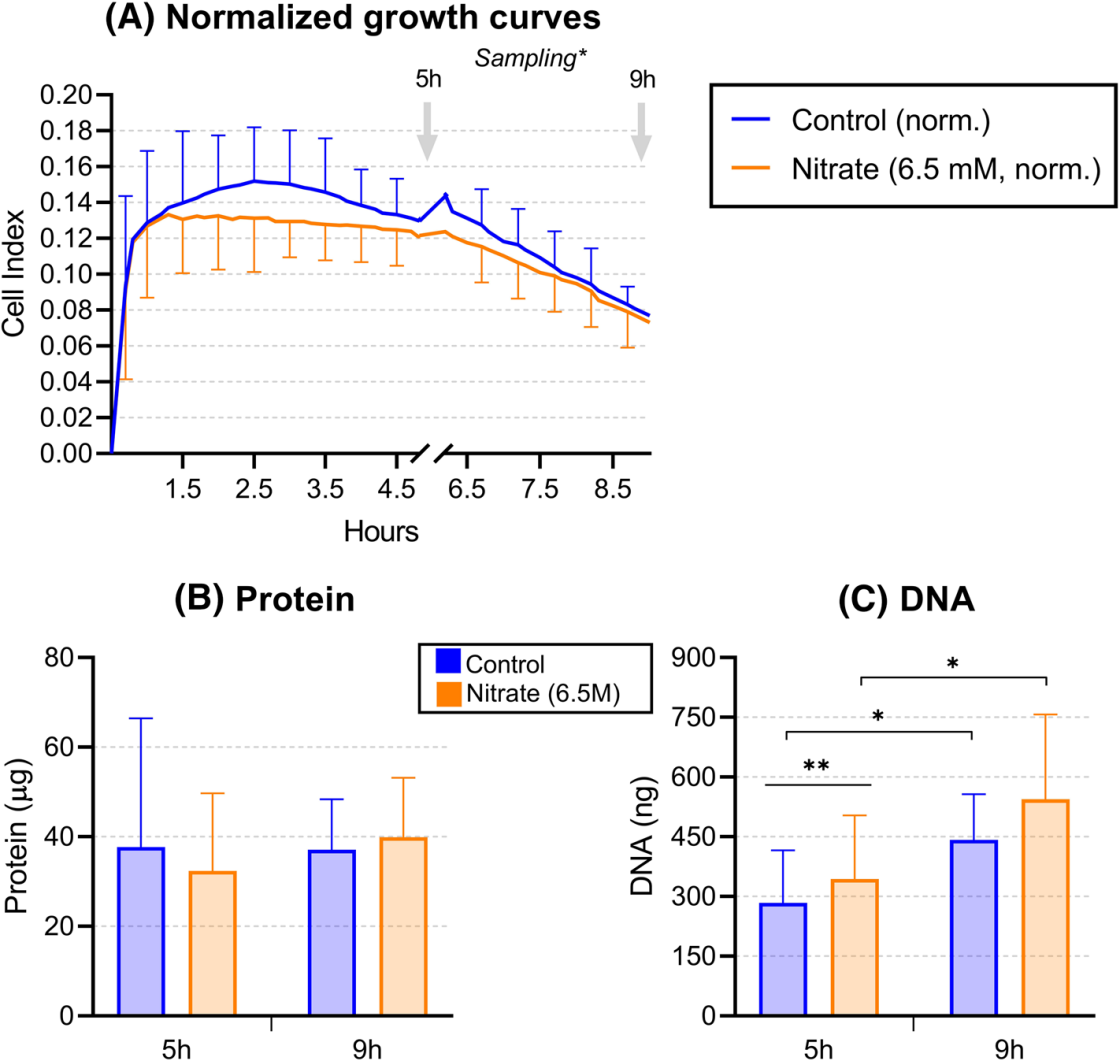
Effect of nitrate on biofilm formation. Biofilms were grown with saliva as inoculum in a 6.5 mM nitrate condition (orange) and a control condition (blue). (**A**) Plot shows averages ± SE of biofilm mass, measured by Cell Index values, as indicated by impedance measurements, after normalization (norm.) with microorganisms-free filtered saliva. Measurements were taken every 10 min. Error bars are only shown at half an hour intervals for clarity. Sampling: samples were collected at 5 h and 9 h for different measurements (grey arrows). (**B**), (**C**) Protein and DNA quantification of the biofilms harvested at 5 h and 9 h. Bars represent averages of 12 donors with their corresponding standard deviations. *p < 0.05, **p < 0.01 according to a Wilcoxon test.

The addition of 6.5 mM nitrate (i.e., 403 mg/L) did not show significant changes in real-time impedance measurements of biofilm formation compared to the control condition (Fig. 1A). In agreement with this, total protein measurements of formed biofilms did not differ significantly between the different conditions (Fig. 1B). There was a positive correlation between DNA and protein of the communities (R = 0.62, p < 0.01). The amount of DNA, however, was 21% higher in the nitrate condition at 5 h (p < 0.01, Fig. 1C), suggesting that the number of cells could be higher under nitrate supplementation or that there is an increase in extracellular DNA. Additionally, 56% and 58% more DNA were detected in the control and nitrate conditions, respectively, at 9 h compared to 5 h (both p < 0.05).

### Changes in nitrate, nitrite, ammonium, lactate and pH during biofilm growth

**Figure 2:**
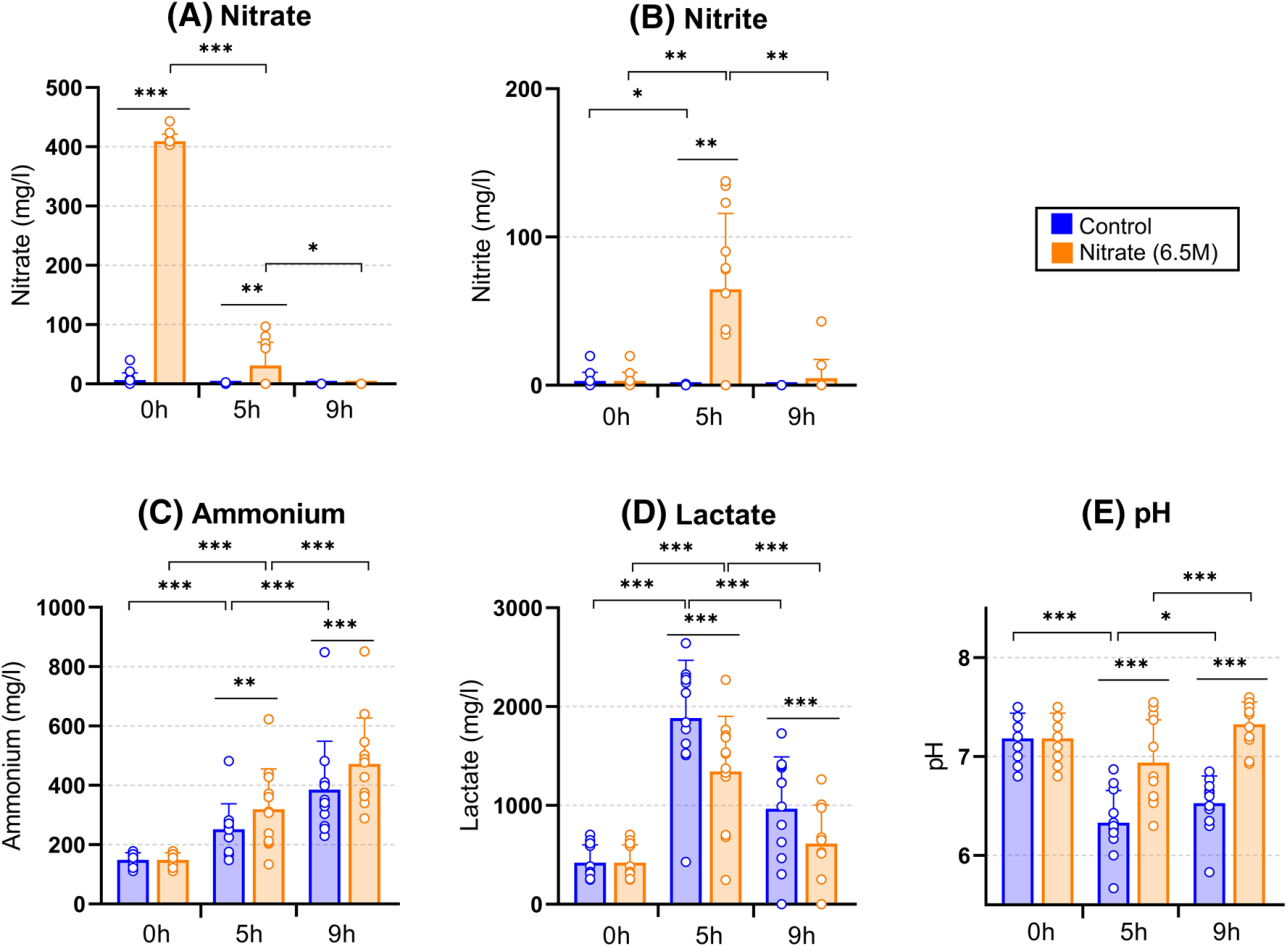
Effect of nitrate supplementation on in vitro oral biofilm metabolism. Barplots show averages and standard deviations of measurements in supernatant samples from 12 donors under 6.5 mM nitrate (orange) and control (blue) conditions at different times of biofilm growth (0 h is the 1:1 mixture of saliva and medium, and 5 h and 9 h are supernatant measurements after in vitro growth). (**A**) Nitrate (mg/L), (**B**) nitrite (mg/L), (**C**) ammonium (mg/L), (**D**) lactate (mg/L) and (**E**) pH. ***p < 0.005, **p < 0.01, *p < 0.05, according to a Wilcoxon test.

Mixtures of saliva and BHI medium with or without 6.5 mM nitrate before growth (0 h) and the supernatants after 5 h and 9 h of growth were analyzed. At baseline (0 h), there were differences in the measured parameters between donors due to person-specific saliva properties (Fig. 2A–E; for measurements in individual donors, see Supplementary Spreadsheet).

In the condition with an additional 6.5 mM nitrate (i.e., 403 mg/L), most nitrate was used up after 5 h (Fig. 2A): in 7 individuals there was no nitrate detectable after 5 h, while for the other 5 donors, 76–85% of the nitrate had been reduced. In accordance with this, nitrite increased from an average of 1.64 mg/L at 0 h to 64.68 mg/L at 5 h. However, at 9 h the nitrite levels had dropped significantly, indicating further reduction to other compounds (Fig. 2B).

After 5 h, the mean of ammonium had increased 1.72 × in the control condition and 2.21 × in the nitrate condition compared to 0 h (both p < 0.005). Ammonium was significantly higher in the nitrate condition compared to the control conditions at 5 h (p < 0.01, Fig. 2C) and 9 h (p < 0.005).The mean of lactate increased notably after 5 h in both conditions compared to 0 h (4.74 × in the control condition and 3.40 × in the nitrate condition, both p < 0.005). After 9 h, the lactate had decreased in both conditions compared to 5 h (p < 0.005), indicating lactate was being metabolized. Importantly, lactate was significantly lower in the nitrate condition compared to the control condition at 5 h and 9 h (p < 0.005, Fig. 2D). There was a negative correlation between lactate and ammonium at 9 h that was more evident in the control condition (at 9 h, r = − 0.87, p < 0.005 in control and r = − 0.71, p < 0.05 in nitrate condition, Tables 1 and 2).

**Table 1:** Correlations of physiological parameters in control condition after 5 h and 9 h (n = 12).

Control condition	Control condition					
	Ammonium (mg/L)		Lactate (mg/L)		pH	
	5 h	9 h	5 h	9 h	5 h	9 h
**Ammonium (mg/L)**						
5 h	1.000	0.860***	− 0.566	− 0.769***	0.217	− 0.147
9 h	0.860***	1.000	− 0.720**	− 0.874***	0.350	0.196
**Lactate (mg/L)**						
5 h	− 0.566	− 0.720**	1.000	0.860***	− 0.203	− 0.434
9 h	− 0.769***	− 0.874***	0.860***	1.000	− 0.196	− 0.399
**pH**						
5 h	0.217	0.350	− 0.203	− 0.196	1.000	0.427
9 h	− 0.147	0.196	− 0.434	− 0.399	0.427	1.000

*p < 0.05, **p < 0.01, ***p < 0.005.

**Table 2:** Correlations of physiological parameters in nitrate condition after 5 h and 9 h (n = 12).

Nitrate condition	Nitrate condition							
	Nitrate (mg/L)^a^	Nitrite (mg/L)^a^	Ammonium (mg/L)		Lactate (mg/L)		pH	
	5 h	5 h	5 h	9 h	5 h	9 h	5 h	9 h
**Nitrate (mg/L)**^a^								
5 h	1.000	0.322	− 0.717**	**− **0.530	0.405	0.711**	**− **0.336	**− **0.016
**Nitrite (mg/L)**^a^								
5 h	0.322	1.000	− 0.641*	**− **0.528	0.655*	0.578*	− 0.822***	**− **0.394
**Ammonium (mg/L)**								
5 h	− 0.717**	− 0.641*	1.000	0.888***	**− **0.573	− 0.694*	0.651*	0.035
9 h	**− **0.530	**− **0.528	0.888***	1.000	− 0.594*	− 0.708*	0.641*	0.091
**Lactate (mg/L)**								
5 h	0.405	0.655*	− 0.573	− 0.594*	1.000	0.753***	− 0.571	− 0.308
9 h	0.711**	0.578*	− 0.694*	− 0.708*	0.753***	1.000	− 0.461	− 0.070
**pH**								
5 h	− 0.336	− 0.822***	0.651*	0.641*	− 0.571	− 0.461	1.000	0.588*
9 h	− 0.016	− 0.394	0.035	0.091	− 0.308	− 0.070	0.588*	1.000

^a^After 9 h, there was no nitrate and nitrite detected in supernatants of 12/12 and 9/12 donors, respectively.

*p < 0.05, **p < 0.01, ***p < 0.005.

In accordance with a higher amount of ammonium production and lower amounts of lactate, pH was significantly higher in the nitrate condition at 5 h and 9 h (both, p < 0.005, Fig. 2E). In this regard, the pH dropped significantly after 5 h compared to 0 h in the control condition (p < 0.005), but not in the nitrate condition (p = 0.056). Interestingly, at 5 h, there was a negative correlation between pH and nitrite in the nitrate condition (r = − 0.82, p < 0.005, Table 2): individuals with 0 mg/L nitrite, possibly all used up, had the highest pH. Likewise, in the nitrate condition, ammonium correlated negatively with nitrite at 5 h (r = − 0.64, p < 0.05), indicating that part of the nitrite was further reduced to ammonia. In agreement with this, there was a negative correlation between the nitrate reduction capacity of communities (determined by the nitrate left after 5 h in the nitrate condition) and ammonium detected in both the nitrate and the control conditions (both r = − 0.717, p < 0.01, Table 2 and Supplementary Table 1). This suggests that communities with the best nitrate reduction capacity produced most ammonia (in the presence or absence of nitrate), indicating a possible link between the two processes.

### The effect of different nitrate concentrations on acidification by sugar metabolism

**Figure 3:**
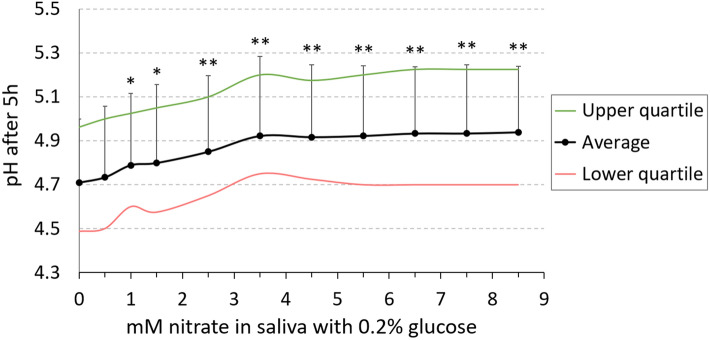
Salivary acidification is inhibited by nitrate. Saliva of 9 donors was incubated for 5 h with 0.2% glucose and a given concentration of nitrate (0.5–8.5 mM), which is within the physiological range of human saliva. In this plot, averages (black dots) with standard deviations, upper quartiles (green line) and lower quartiles (red line) are shown. All the different concentrations of nitrate were compared with 0 mM nitrate and significance was marked with * for p < 0.05 and ** for p < 0.01 according to a Wilcoxon test.

To investigate if nitrate would have an effect on salivary acidification by sugar without the presence of culture medium, unstimulated saliva was incubated with 0.2% glucose and a concentration range of nitrate from 0.5–8.5 mM during 5 h (Fig. 3). The salivary pH before growth was 7.17 (SD 0.41). After 5 h of incubation with 0.2% glucose without nitrate, the pH dropped to pH 4.71 (SD 0.29, LQ 4.49, UQ 4.96). All nitrate concentrations from 1 mM to 8.5 mM resulted in a significantly higher pH after 5 h compared to 0 mM nitrate (p < 0.05 for 1 mM and 1.5 mM, p < 0.01 for higher concentrations up to 8.5 mM). Interestingly, 3.5 mM nitrate resulted in pH 4.92 (SD 0.36, LQ 4.75, UQ 5.2) and the pH levels did not further increase significantly when adding more nitrate.

### Nitrate strongly affects biofilm composition

**Figure 4:**
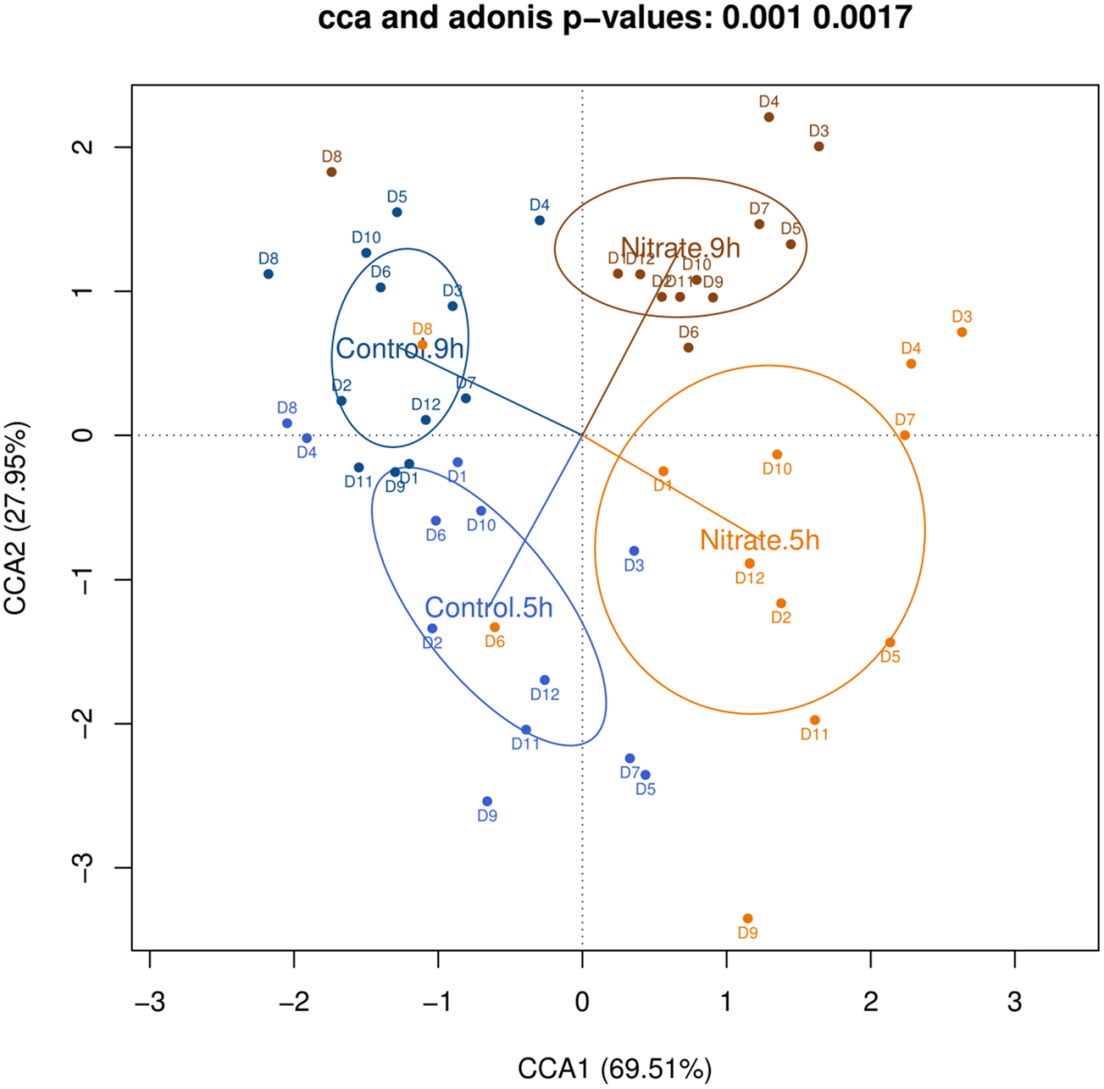
Effect of nitrate on oral biofilm composition at 5 h and 9 h of in vitro growth. Both Adonis and CCA p-values suggest statistically significant differences in bacterial composition on a genus-level between the control and nitrate conditions at 5 h and 9 h (for two-group comparisons, see Supplementary Figure 1). In this plot, the first constrained component clearly separates the two experimental conditions (control and nitrate), whereas the second one reflects variability due to time (5 h and 9 h), showing that both biofilm formation time and nitrate influence bacterial composition.

The addition of nitrate had a significant effect on the bacterial composition of in vitro oral communities, explaining a large proportion of data variability regardless of biofilm sampling time (Fig. 4). The control and nitrate conditions differed significantly at 5 h and 9 h (individual CCA and Adonis p-values ≤ 0.005, Supplementary Figure 1). The CCA p-value between the control condition at 5 h and 9 h was not significant, but the Adonis p-value was (< 0.05). The difference between the nitrate condition at 5 h and 9 h was significant (CCA and Adonis p-value < 0.05), indicating that oral communities can change rapidly (in a matter of a few hours) under certain conditions, especially in the presence of nitrate.

**Figure 5:**
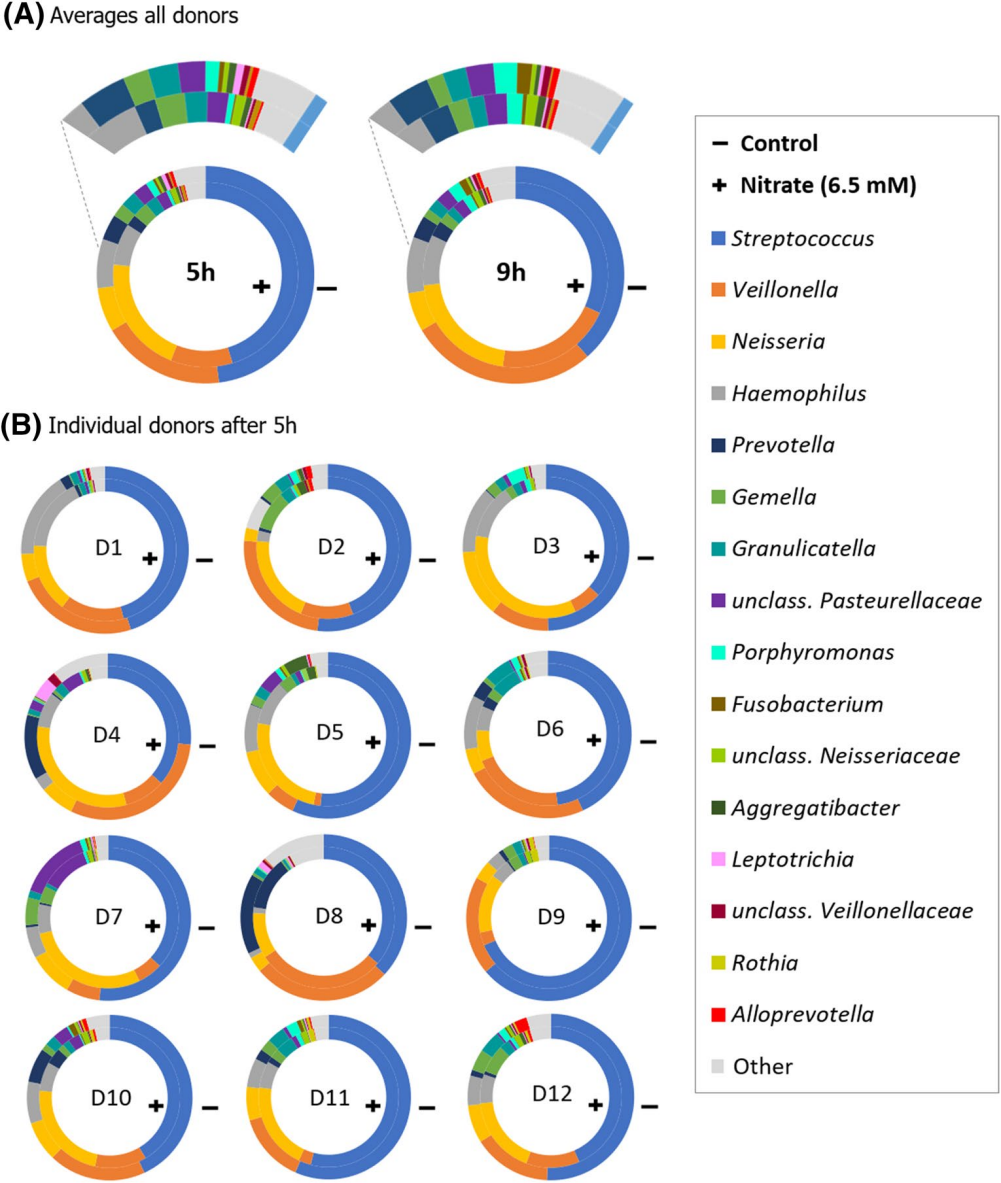
Bacterial composition in oral biofilms as determined by 16S rRNA sequencing. (**A**) Ring charts of the average relative abundance of genera in biofilms of 12 donors at 5 h and 9 h of growth. Above the ring charts, there is a zoom-in showing the low-abundance genera between *Haemophilus* (dark grey) and *Steptococcus* (blue). (**B**) Relative abundance of genera in biofilms of individual donors at 5 h. In this figure, the outer rings are the control condition (−) and the inner rings the condition with 6.5 mM nitrate (+). Genera at < 0.1% abundance are indicated as “Other” for clarity. The genera are sorted based on their maximum average abundance in one of the conditions from most to least abundant. Data for the inoculum and 9 h can be found in Supplementary Figure 2.

The relative abundances of individual donors at 5 h and 9 h were plotted as percentages in Fig. 5B and Supplementary Figure 2B, respectively (individual data can be found in the Supplementary Spreadsheet). Based on medians, in the control condition, the five most common genera after 5 h of biofilm growth were *Streptococcus* (50.17%), *Veillonella* (19.14%), *Neisseria* (6.62%), *Haemophilus* (6.16%) and *Granulicatella* (1.94%). In the nitrate condition, the most abundant genera after 5 h were *Streptococcus* (43.81%), *Neisseria* (20.27%), *Veillonella* (10.63%), *Haemophilus* (6.64%) and *Gemella* (2.17%). After 9 h, *Streptococcus* remained the dominant genus (38.94% and 32.78% in control and nitrate conditions, respectively) followed by *Veillonella* (28.85%), *Haemophilus* (8.75%), *Neisseria* (6.27%) and *Porphyromonas* (1.77%) in the control condition and by *Neisseria* (20.55%), *Veillonella* (19.53%), *Haemophilus* (8.22%) and *Gemella* (1.48%) in the nitrate condition. The saliva used as inoculum of 4 randomly selected donors was sequenced (Supplementary Figure 2A) and comparable dominant genera were found as in the in vitro biofilms (Fig. 5A and Supplementary Figure 2B): with a similar percentage of *Streptococcus* (43.88%) on the first place in the inoculum and then *Veillonella* (7.19%), *Neisseria* (6.97%), *Gemella* (6.44%) and *Granulicatella* (5.27%).

**Figure 6:**
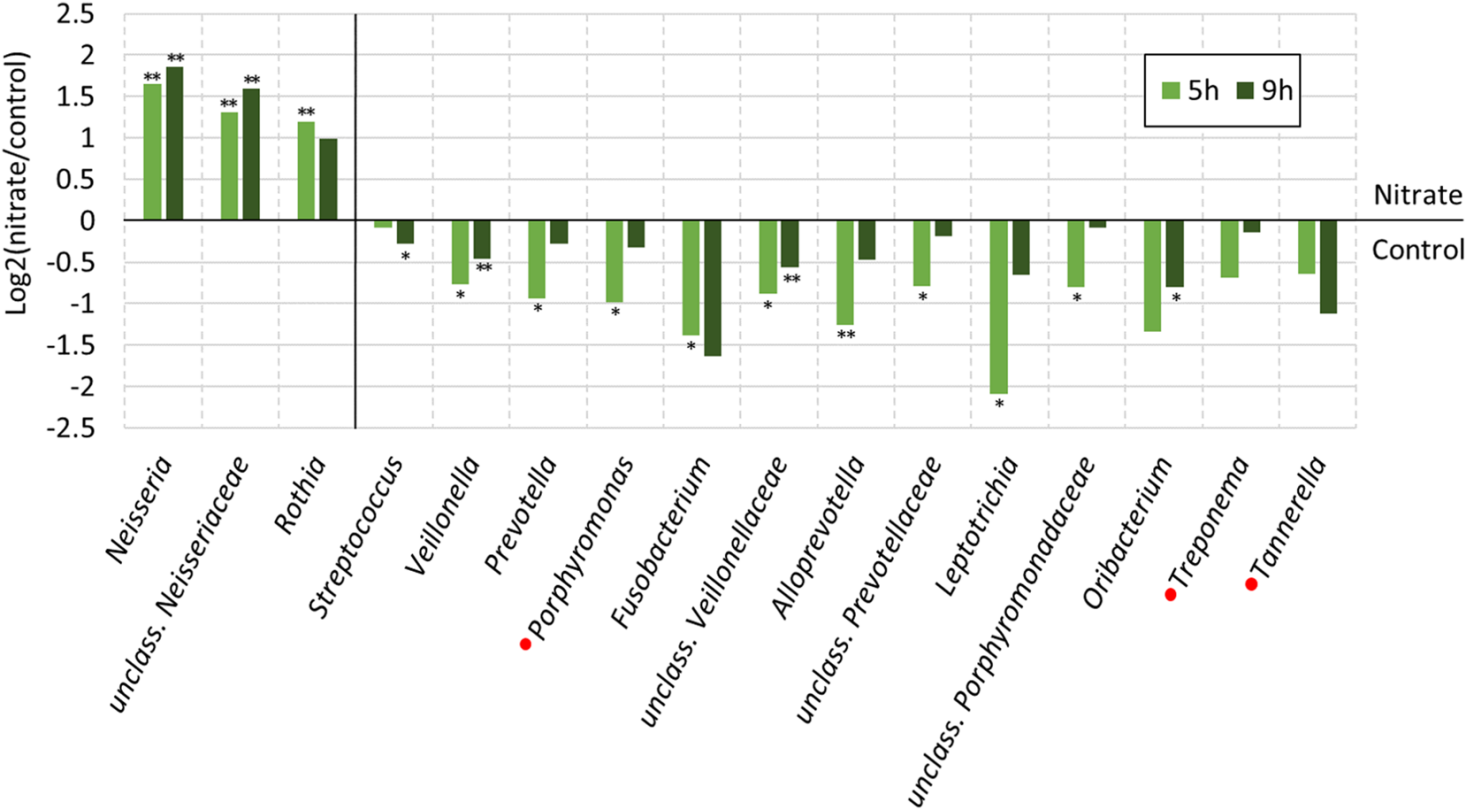
Changes in biofilm bacterial composition under nitrate conditions. Bar graphs show the log2 value of the ratio [average abundance nitrate condition]/[average abundance control condition] of 12 donors. Genera shown are those significantly different between the 6.5 mM nitrate and control conditions at 5 h or 9 h and, additionally, *Treponema* and *Tannerella*—two clinically relevant genera—were added. The genera that are higher in the nitrate condition are listed first and sorted by their highest average abundance in one of the conditions. After the vertical black stripe, the genera that are lower in the nitrate condition are listed, sorted by their highest average abundance in one of the conditions from highest to lowest (all taxa after unclass. *Veillonellaceae* had an average abundance of < 0.5% in all conditions). Red circles are placed before the genera of periodontitis-associated “red-complex” bacteria. unclass. = unclassified (only shown at family level); *adjusted p < 0.05 and **adjusted p < 0.01 between the control and nitrate conditions, according to a Wilcoxon test (Supplementary Spreadsheet).

To study the effect of nitrate supplementation, significant changes in genera between the nitrate and the control conditions at 5 h or 9 h were analyzed (see Supplementary Spreadsheet for all p-values). The lower abundance of *Veillonella* (a bacterium that uses lactate as carbon source) in the nitrate condition compared to the control condition was significant at 5 h and 9 h (p < 0.05 and p < 0.01, respectively, Fig. 6) as well as the lower percentage of *Streptococcus* (a genus known to produce lactate) at 9 h (p < 0.05). The genus *Prevotella* at 5 h was lower in the control condition (1.51% compared to 0.78% in the nitrate condition, p < 0.05). No significant differences were observed between *Haemophilus and Gemella* in the two conditions. The dominant OTUs within each genera are listed in Supplementary Tables 2 and 3.

The 3.1 × increase at 5 h and 3.3 × at 9 h of the median of *Neisseria* in the nitrate condition compared to the control condition were significant (both p < 0.01, Fig. 6). Identified *Neisseria* OTUs included *N. flavescens*, *N. subflava*, *N. bacilliformis*, and *N. elongata*. *Rothia*, another nitrate-reducing genera dominated by an OTU classified as *R. aeria* or *R. dentocariosa*, was at low abundance (i.e., median in all conditions 0.14%, range 0.01–1.7%), but the median was 2.9 × higher in the nitrate condition (0.35%) than in the control condition at 5 h (0.12%, p < 0.01). A third nitrate-reducing bacterium, *Kingella*, was present at low abundance but also at higher levels in the nitrate condition. However, the difference was not significant (p = 0.15).

Other genera significantly lower in the nitrate condition were periodontitis and/or halitosis-associated *Porphyromonas* (including the OTU *P. endodontalis* or *oral taxon 285*), *Fusobacterium* (including *F. periodonticum* and *F. nucleatum*), *Leptotrichia* (including the OTU *L. wadei* or oral taxon 417, and *L. hongkongensis*) and *Avoprevotella* (including *A. rava* and *A. tannerae*) at 5 h (p < 0.05), and the caries-associated *Oribacterium* (including *O. parvum* and *O. sinus*) at 9 h (p < 0.05). Decreases were also observed for the periodontitis-associated “red complex” bacteria *Tannerella* and *Treponema* (Fig. 6), but these differences were not significant using Wilcoxon adjusted p-values.

When using ANCOM-II adjusted p-values (Supplementary Figure 3 and Supplementary Spreadsheet), the significant changes in genera between the control and nitrate conditions were consistent (i.e., genera that differed significantly between the nitrate and control conditions at 5 h and/or 9 h using Wilcoxon adjusted p-values, still differed significantly at 5 h and/or 9 h using ANCOM-II adjusted p-values). However, several additional genera decreased significantly in the nitrate condition compared with the control condition when using ANCOM-II. These were *Tannerella* at 9 h, and *Granulicatella*, *Atopobium*, *Actinomyces*, *Lachnoanaerobaculum* and *Dialister* at 5 h.

In the nitrate condition, *Veillonella* and *Actinomyces* correlated negatively with pH at 9 h (r = − 0.77 and − 0.72, respectively, both p < 0.05, Supplementary Figure 4). In contrast, *Neisseria* correlated positively with pH at 9 h in the nitrate condition (r = 0.84, p < 0.01), as well as at 5 h in the control condition (r = 0.75, p < 0.05).

## Discussion

**Figure 7:**
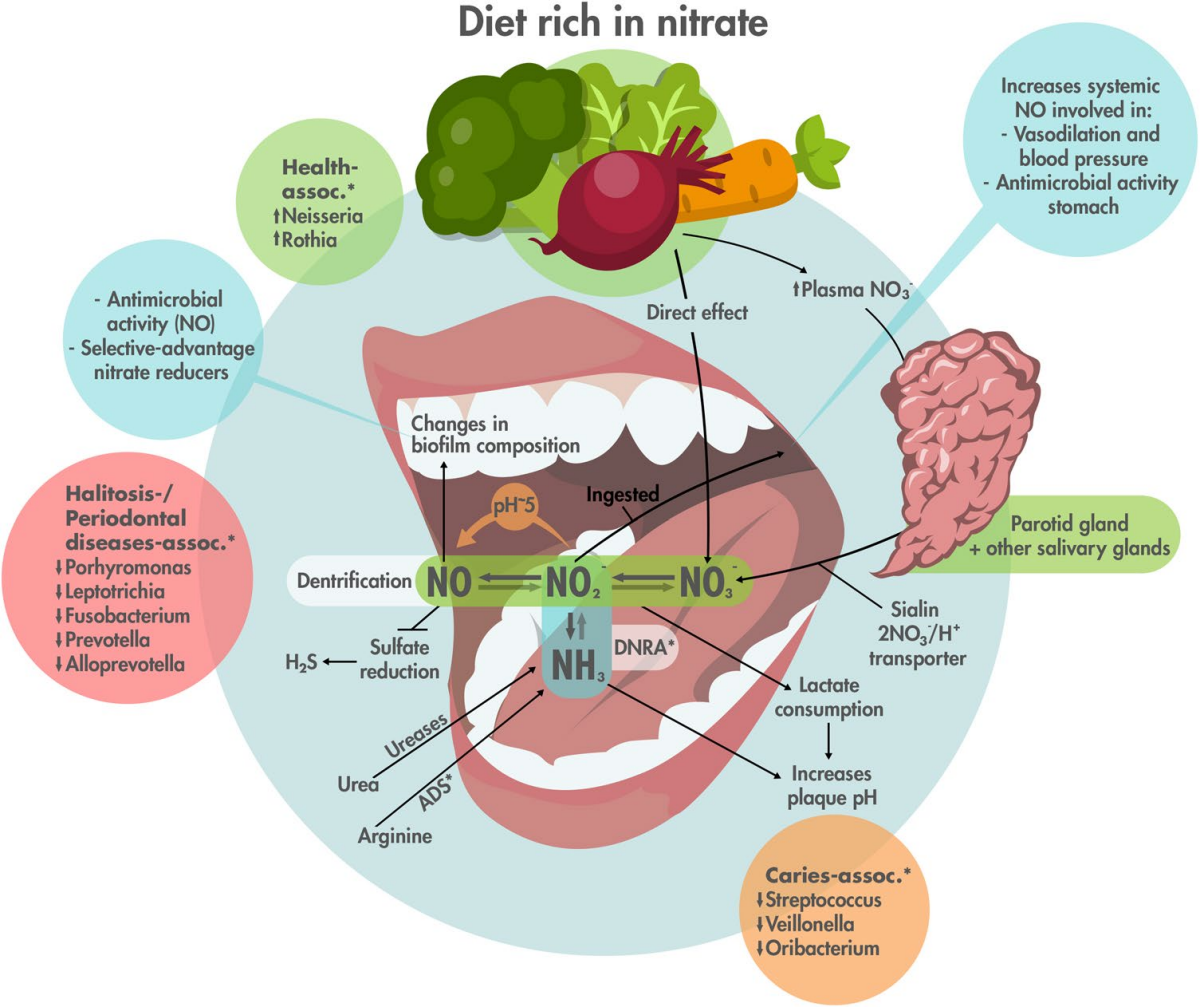
Overview of potential effects of nitrate inside the oral cavity. This graphical summary of the discussion is based on this study and current literature. Nitrate (NO_3_^−^) from food, such as vegetables (e.g., leafy greens, beetroots and carrots) and fruits, enters the blood stream and plasma nitrate is concentrated into saliva by sialin transporters in the salivary glands. There is also a direct effect of nitrate when the foods are chewed or pass through the mouth. Nitrate is reduced into nitrite (NO_2_^−^) and further to nitric oxide (NO) by denitrifying oral bacteria. Nitric oxide is an antimicrobial molecule that could limit the growth of certain species (e.g., periopathogenic species have been shown to be sensitive to NO) and thereby affecting the composition of oral biofilms. Additionally, at a pH of 5 and lower, acidic decomposition of nitrite to nitric oxide takes place (orange arrow), which could stimulate the antimicrobial effect when the pH drops due to sugar fermentation. Nitrite can also be reduced to ammonia (NH_3_) by the bacterial Dissimilatory Nitrate Reduction to Ammonium (DNRA) pathway, increasing the local pH. Additionally, nitrate-reducing species can use lactate as a carbon source, which further prevents a drop in pH. Other bacterial enzymes that lead to ammonia production are ureases using urea as a substrate and arginine deiminase system (ADS) enzymes using arginine. Denitrification is more energy efficient than sulfate reduction and, therefore, the presence of nitrate should limit hydrogen sulfide (H_2_S) production. In our study, we observed an increase of the health-associated genera *Neisseria* and *Rothia*, while caries-associated genera *Streptococcus*, *Veillonella* and *Oribacterium* decrease, as well as the anaerobic periodontal diseases- and halitosis-associated genera *Porphyromonas*, *Fusobacterium*, *Leptotrichia*, *Prevotella*, and *Alloprevotella*. It should be noted that these genera may also contain health-associated representatives. However, generally, the total abundance of these genera increases in the associated diseases. *assoc. associated genera, *ADS *arginine deiminase system.

The salivary glands contain electrogenic sialin 2NO_3_^−^/H^+^ transporters, which after a nitrate-containing meal increase salivary nitrate to millimolar levels, resulting in elevated nitrate concentrations over many hours^43^. The data presented in the current manuscript indicate that nitrate supplementation at the physiological levels found in saliva is able to prevent or reduce bacterial dysbiosis and stimulate eubiosis by elevating health-associated genera and reducing the levels of disease-associated bacteria. In addition, our work provides some of the mechanisms underlying the potential beneficial effect of nitrate for oral health, including lactate consumption or ammonia-mediated pH buffering (Fig. 7).

It is surprising that nitrate metabolism in the oral cavity has scarcely been studied as a potential factor influencing biofilm composition and activity, as nitrate is an important ecological factor influencing the composition and functioning of microbial communities in natural environments^6,44^. There is evidence suggesting that nitrate reduction is also relevant for the oral cavity, where nitrate appears to lead to health-associated changes in the oral microbiota. For instance, nitrate metabolism has been associated with lower caries abundance^11^ and in a recent clinical study, gingival inflammation in patients with chronic gingivitis was significantly reduced after 14 days of nitrate intake in the form of lettuce juice^13^.

Our data show that less lactate and more ammonium was produced after 5 h and 9 h of in vitro oral biofilm growth in the presence of 6.5 mM nitrate, and, accordingly, the pH was higher than in the control condition without nitrate (all p < 0.01). Additionally, there was a strong negative correlation between lactate and ammonium produced after 9 h, which was more evident in the nitrate condition. This supports the hypotheses by Li et al. (2007)^12^ that alkali production and lactate consumption by nitrate-reducing communities limit a drop in pH when carbohydrates are fermented. In vivo this could potentially reduce the time that the dental tissue is under demineralizing pH^45^. In our study, we found that nitrate concentrations from 1 mM prevented salivary acidification due to glucose fermentation, while no additional pH buffering was observed for concentrations above 3.5 mM. Li et al.^12^ only used a nitrate concentration of 1.5 mM in modified saliva samples and also observed that nitrate prevented a drop in pH due to sugar fermentation by oral microorganisms under anaerobic conditions, but not under aerobic conditions. In addition, Burleigh et al. (2020)^46^ observed an increase in salivary pH after 7 days of nitrate-rich beetroot juice supplementation, which could be explained by the mechanisms detected in our in vitro study.

In our study, biofilm growth curves and protein levels were not significantly different between the control and the nitrate condition. Notably, biofilms grown with nitrate contained several times higher levels of *Neisseria* and *Rothia*, which are nitrate-reducing genera^47,48^, already after a short period of 5 h (p < 0.01) and *Neisseria* remained significantly higher after 9 h (p < 0.01). This included an increase of total abundance of *Rothia mucilaginosa* and *Neisseria flavescens*, which was also observed in vivo by Velmurugan et al. (2016)^14^ in saliva after 6 weeks of daily beetroot juice intake. Vanhatalo et al. (2018)^15^ showed that 10 days of daily beetroot intake increased *Rothia* and *Neisseria*, while decreasing *Veillonella* and *Prevotella*, in saliva, which is in accordance with our results in newly formed in vitro oral biofilms. Putting these results together, it appears that species of *Rothia* and *Neisseria* have a selective advantage in the presence of nitrate.

In the majority of recent sequencing studies, different *Neisseria* and *Rothia* species have been associated with disease-free individuals. For example, *Rothia* spp. and *Neisseria* spp. were more abundant in subgingival plaque of periodontally healthy individuals compared to patients with periodontitis^49–52^. Apart from relative abundance, also the prevalence of *Rothia* and *Neisseria* is higher in healthy subgingival plaque compared to periodontitis samples^53^.

Periodontitis is a chronic and destructive inflammation of the gingiva and can result from repeated or long-lasting episodes of gingivitis (i.e., reversible inflammation of the gingiva). In one study, the genus *Neisseria* correlated with anti-inflammatory mediators and was associated with a better recovery of the gingiva after experimental gingivitis^54^. In another study, *Rothia aeria* negatively correlated with inflammatory cytokines IL-17 and TNF-α^55^, whereas *Rothia dentocariosa* has been found to induce TNF-α production in a human cell line in vitro^56^. Given the high dynamic nature observed for many oral bacteria, nitrate-reducing isolates that show promising features as oral probiotics should be individually tested to confirm their systemic safety^57^.

Additionally, both *Rothia*^58,59^ and *Neisseria*^58,60^ species were more abundant in supragingival plaque of caries-free individuals compared to individuals with active caries. In a recent study, *Rothia dentocariosa* was also found to be more abundant on the tongue of halitosis-free individuals compared to halitosis patients^61^. In conclusion, *Neisseria* and *Rothia* are associated with oral disease-free individuals and an increase in these genera can be considered a positive change in the microbiota related to general oral health (i.e., eubiosis).

The DNA of biofilms in our study was 21% higher in the nitrate condition compared to the control condition after 5 h (p < 0.01), but not significantly different after 9 h. The small increase in DNA after 5 h could have resulted from a higher number of cells from, e.g., *Rothia* and *Neisseria*. Alternatively, the higher DNA amount could be derived from a larger production of extracellular DNA, which has previously been shown to affect biofilm integrity and adhesion^62^. Given that extracellular DNA could affect 16S rRNA sequencing results, in future experiments the samples could be treated with DNAse prior to DNA isolation to remove the effect of this extracellular DNA on bacterial composition assessment. Additionally, possible differences in biofilm properties due to extracellular DNA should be tested.

Regarding disease-related bacterial composition, we observed a decrease in *Veillonella*, *Streptococcus* and *Oribacterium*, which are genera associated with lactate, acidification and caries^63–65^, in the nitrate condition after 9 h (p < 0.05). Future studies performed with longer sequences should focus on species-level analyses, because even when a given genus is generally associated with disease (i.e., a consistent increase in disease is observed when compared with health in different studies), species^66,67^ and probably even strains within species could be associated with health.

Another important observation in our experiments was that periodontal-disease associated *Porphyromonas*, *Fusobacterium, Prevotella, Leptotrichia* and *Alloprevotella* were significantly lower in biofilms grown with nitrate after 5 h (p < 0.05). *Porphyromonas*, *Fusobacterium,* and *Prevotella* contain species of the classic ‘red and orange complexes’ identified by Socransky et al., which are strongly associated with periodontitis^68^. These include *Fusobacterium periodonticum*, *Prevotella intermedia* and *Prevotella nigrescens* that were also identified in our study. Similarly, *Leptotrichia* has a strong association with periodontitis^69^, while very recently it was confirmed that *Alloprevotella* is more abundant in disease as well^67^. Nitric oxide-releasing material and metal oxides have been shown to have antimicrobial activity against several periopathogenic species, including *Porphyromonas gingivalis*, *Prevotella intermedia* and *Fusobacterium nucleatum*^70^. Nitric oxide resulting from nitrate reduction could have killed slow-growing anaerobic bacteria in the inoculum, explaining their decrease after 5 h.

It is interesting to note that *Porphyromonas*, *Fusobacterium*, *Leptotrichia* and *Prevotella* are also associated with halitosis—bad breath resulting from microbial production of volatile sulfur compounds (VSCs)^71^. It must be kept in mind that nitrate is used as a biological agent to treat malodour from sewer networks by limiting microbial VSCs production resulting from sulfate reduction^72^. As long as more energy-efficient denitrification takes place, sulfate reduction is inhibited and this could also be the case in the oral cavity, where sulfate reduction is associated to halitosis^73^ (Fig. 7). The effect of nitrate supplementation on sulfate reduction and halitosis should be tested in future clinical studies.

The metabolism of nitrite and the production of ammonium in our study indicate the presence of Dissimilatory Nitrate Reduction to Ammonium (DNRA) activity by oral species. The observation that nitrite correlated negatively with ammonium and pH at 5 h (biofilms that metabolized all nitrite produced most ammonium and had the highest pH) further supports this. In the nitrate condition, the amount of ammonium after 9 h was 4.75 mM higher than in the control condition. Stoichiometrically, this could account for 73.1% of the 6.5 mM added nitrate, while (part of) the other 26.9% of nitrate may have been denitrified into nitric oxide and other nitrogenous products. It is unlikely that nitrate was used in assimilatory pathways as low concentrations of ammonium, which were present in all cultures, inhibit nitrate assimilation^44,74^. Interestingly, several environmental conditions, including pH and the protein:carbohydrate ratio^75^, direct the conversion of nitrate into ammonia or nitric oxide, and their role in regulating nitrate reduction in the oral cavity should be further investigated.

Arginine has received much attention as a prebiotic that is converted by certain oral microorganisms into ammonia, increasing the local pH and thereby having an anti-caries effect *in vivo*^76,77^. Urease activity by some oral bacteria has also been shown to buffer acidic pH by ammonia production and was shown to correlate with caries status^78,79^. We provide evidence that nitrate could have a similar effect and suggest that all three metabolic activities (i.e. arginine deaminase, urease and nitrate reduction) are considered when estimating the oral biofilm’s pH buffering capacity.

### Nitrate: from disease-associated to neutral compound with health benefits

Nitrate has had a bad reputation for decades, because under certain conditions, its bacterial reduction product, nitrite, can react with other molecules and form potentially carcinogenic N-nitroso compounds (e.g., nitrosamine)^80,81^. This has been reported on processed meats, where nitrate salts are added as preservatives, resulting from bacterial and chemical reactions over time. However, humans obtain more than 80% of dietary nitrate from vegetables, which is a food group unequivocally associated with health benefits^4^, longevity and lower prevalence of diseases^5^, including cancer^82^. Anti-oxidants in fruits and vegetables prevent the formation of N-nitroso compounds from nitrite and stimulate the formation of nitric oxide^83,84^. In relation to this, different safety agencies stated that epidemiological studies do not suggest that nitrate intake from diet or drinking water is associated with increased cancer risk^85^ (reviewed by Lundberg et al., 2018^4^. In contrast, evidence has accumulated that the oral microbiota-dependent increase of systemic nitric oxide resulting from dietary nitrate can have several beneficial cardio-metabolic effects^4^.

The current acceptable daily intake (ADI) of nitrate is 3.7 mg/kg of body weight (222 mg for an adult of 60 kg). In recent clinical studies focusing on cardiovascular effects, which observed an increase in *Neisseria* and *Rothia* in saliva, high daily doses of nitrate were given to individuals in the form of beetroot juice (i.e., 372–770 mg per day, which is 1.7–3.5 × the ADI) during periods of 1–4 weeks^14,15,46^. In our study, we show that a single dose of 101 µg nitrate to obtain 6.5 mM in a volume of 250 µL was enough to increase these nitrate-reducing genera in a short period of 5 h. To draw a parallel to the oral cavity, where volumes of around 0.5–1 mL of saliva are often found, topical doses far below the ADI would suffice to maintain a 6.5 mM concentration over time.

## Conclusions

The results in this study showed that nitrate caused rapid structural and functional shifts in oral communities in vitro that would be of benefit to the human host. Based on our results, we conclude that nitrate could be an ecological factor stimulating health-associated oral genera, with the potential to decrease caries-, periodontitis- and halitosis- associated genera (Fig. 7). Additionally, we conclude that nitrate metabolism provides resilience to acidification resulting from sugar metabolism, by increasing lactate consumption and ammonia production, and future studies should test this possibility in vivo.

In biofilms grown with nitrate, *Veillonella*—a genus that uses lactate as carbon source—correlated negatively with pH and *Neisseria* positively. Due to the high in vivo prevalence of *Neisseria* and *Rothia* in different oral habitats, we argue that these genera could have essential roles in maintaining a healthy symbiotic relationship between the oral microbiota and the host by the reduction of salivary nitrate.

Taking into account our findings and other studies focusing on nitrate and oral health^11–13,16^, we propose that nitrate is a health-associated molecule in the oral cavity. We therefore suggest that nitrate could be used as a prebiotic (e.g., vegetable extracts or nitrate combined with anti-oxidants), and nitrate-containing vegetables tested in dietary interventions, in order to stimulate eubiosis or reduce dysbiosis in the oral cavity. Representatives of *Neisseria*, *Rothia* and other nitrate-reducing genera (e.g. *Kingella*) have traditionally been associated with oral health in many studies and we propose that this is, in part, due to their capacity to reduce nitrate. We therefore suggest that certain nitrate-reducing strains could be used as probiotics to stimulate the benefits of nitrate metabolism. We hope that, although preliminary, our in vitro data stimulate further research to test the potential effect of nitrate and nitrate-reducing bacteria on oral health in human subjects.

## Supplementary information

Supplementary Information 1.

Supplementary Information 2.

## Data Availability

The data that support the findings of this study are available from the corresponding author upon reasonable request.
