# Supplementary material for: Nitrate as a potential prebiotic for the oral microbiome
Source: Sci Rep. 2020 Jul 30;10:12895. doi: 10.1038/s41598-020-69931-x (PMC7393384; doi:10.1038/s41598-020-69931-x)
Supplement: Supplementary file 1 — Supplementary Information 1. [file 41598_2020_69931_MOESM1_ESM.pdf]

Nitrate as a potential prebiotic for the oral microbiome

Rosier B.T.*, Buetas E.*, Moya-Gonzalvez E.M., Artacho A., Mira A.#

Department of Health and Genomics, Center for Advanced Research in Public Health, FISABIO Foundation, Avenida de Catalunya 21, 46020 Valencia (Spain)

*First authors

#Corresponding author: mira_ale@gva.es

Supplementary files

Supplementary table 1: correlations of physiological parameters between control and nitrate conditions after 5h and 9h (n = 12)

			Control condition					
			Ammonium		Lactate		pH	
			5h	9h	5h	9h	5h	9h
Nitrate condition	Nitrate[a]	5h	**-0,717****	**-0,710****	0.491	**0,795*****	-0.094	-0.140
	Nitrite[a]	5h	**-0,641***	-0.535	0.401	0.500	-0.563	0.007
	Ammonium	5h	**0,937*****	**0,923*****	**-0,615***	**-0,790*****	0.371	-0.021
		9h	**0,811*****	**0,909*****	**-0,762*****	**-0,734****	0.427	0.077
	Lactate	5h	-0.545	**-0,594***	**0,776*****	**0,734****	-0.566	-0.503
		9h	**-0,718****	**-0,771*****	**0,883*****	**0,932*****	-0.165	-0.277
	pH	5h	0.550	0.553	-0.424	-0.448	**0,792*****	0.172
		9h	-0.007	0.154	0.000	-0.084	**0,776*****	0.517

[a] After 9h, there was no nitrate and nitrite detected in supernatants of 12/12 and 9/12 donors, respectively.

* $p < 0.05$

** $p < 0.01$

*** $p < 0.005$

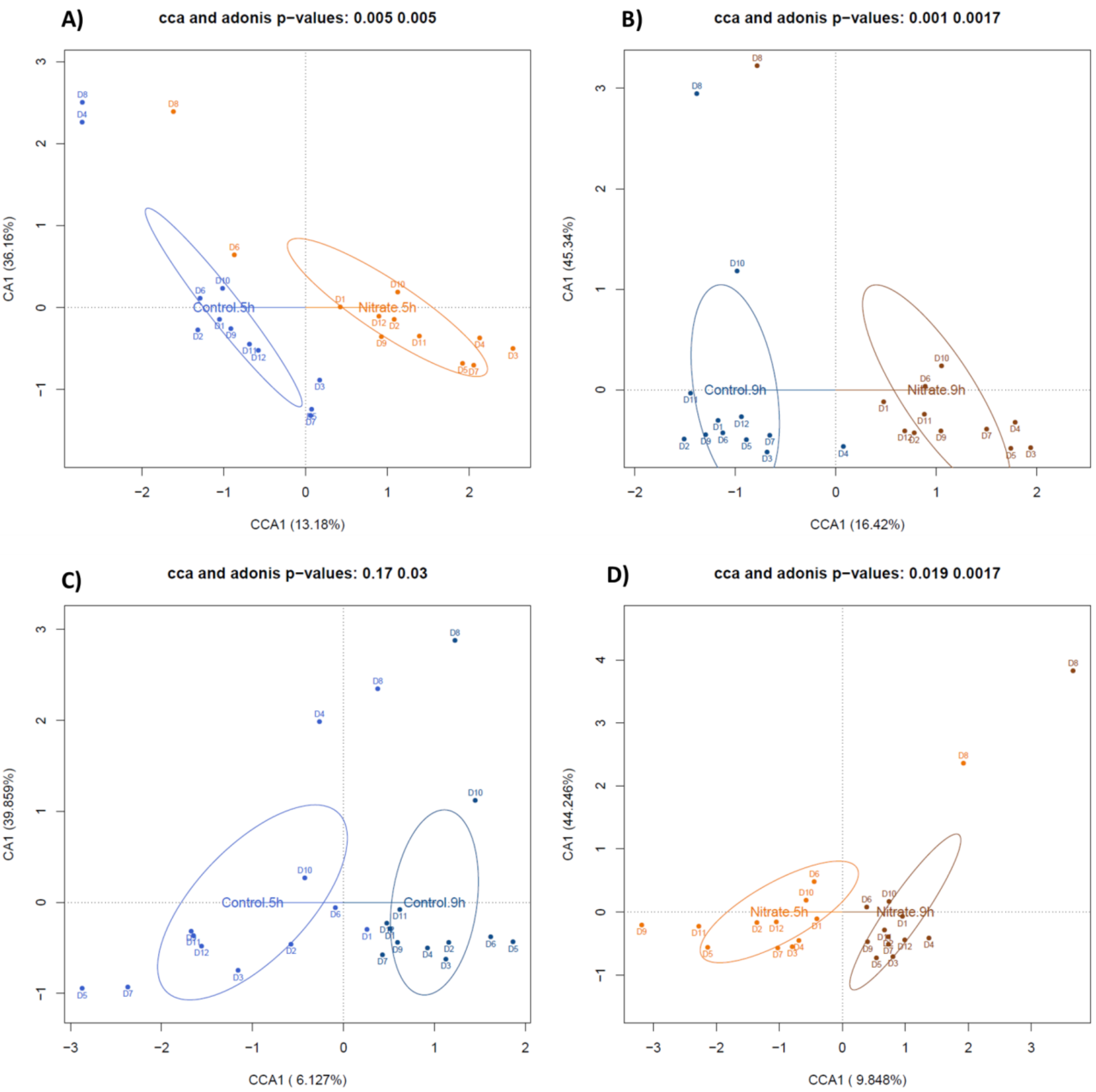

Supplementary Fig. 1: Effect of nitrate on oral biofilm composition at 5h and 9h of *in vitro* growth (two-group comparisons). Both Adonis and CCA p-values are significant between the control and nitrate conditions at 5h (A) and 9h (B). There were also significant differences between 5h and 9h in the control condition (CCA p-value only, C) and nitrate condition (Adonis and CCA p-values, D).

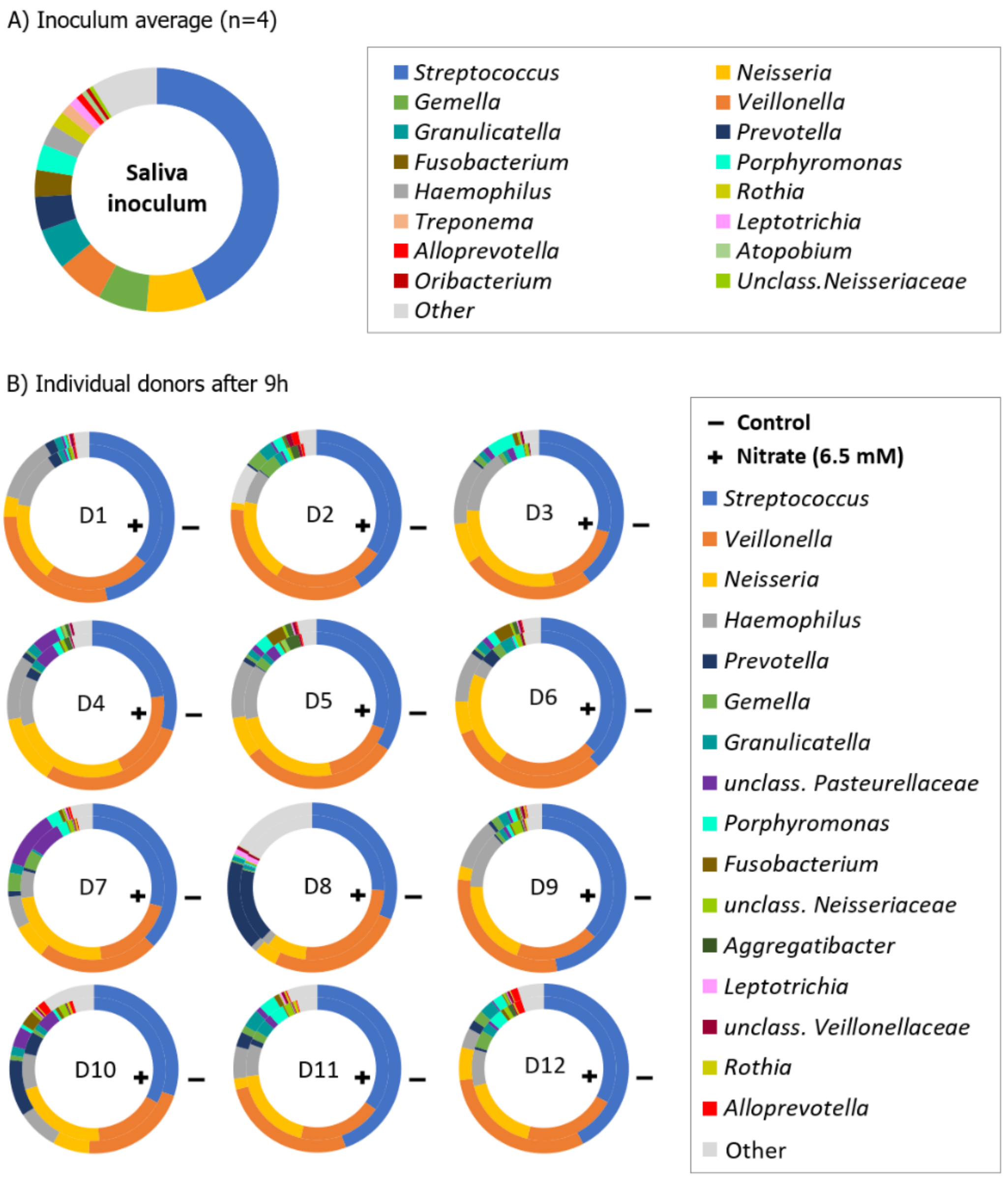

Supplementary Fig. 2: Bacterial composition in inoculum and oral biofilms as determined by 16S rDNA sequencing. A) Ring chart of the average relative abundance of genera in the saliva used as inoculum of donors 2, 3, 4 and 6 (n=4), which were randomly selected. B) Relative abundance of genera in biofilms of individual donors at 9h. In this figure, the outer rings are the control condition (-) and the inner rings the condition with 6.5 mM nitrate (+). Genera at <0.1% abundance are indicated as “Other” for clarity. The genera are sorted based on their maximum average abundance in one of the conditions from most to least abundant.

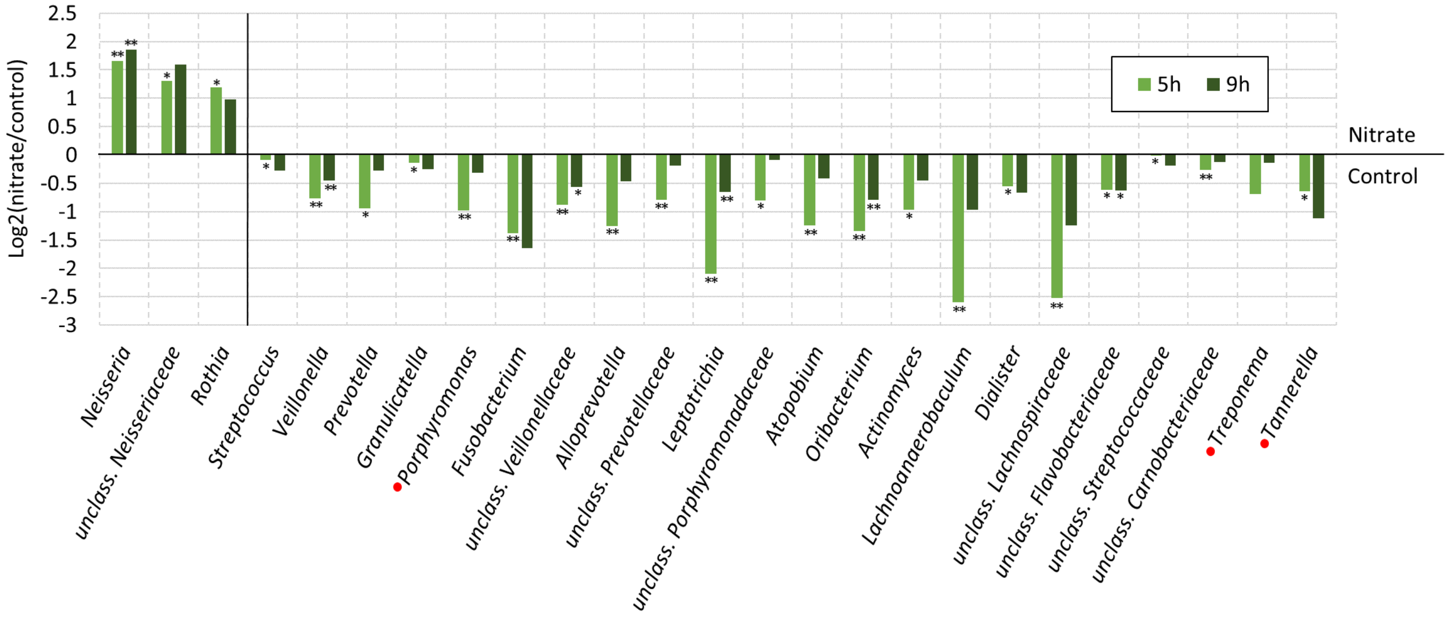

Supplementary Fig. 3: Changes in biofilm bacterial composition under nitrate conditions (based on Standard Compositional Data Analysis, CODA). Bar graphs show the log2 value of the ratio [average abundance nitrate condition]/[average abundance control condition] of 12 donors. Genera shown are those significantly different between the 6.5 mM nitrate and control conditions at 5h or 9h and, additionally, *Treponema*-a clinically relevant genera- was added. The genera that are higher in the nitrate condition are listed first and sorted by their highest average abundance in one of the conditions. After the vertical black stripe, the genera that are lower in the nitrate condition are listed, sorted by their highest average abundance in one of the conditions from highest to lowest (all taxa after unclass. Veillonellaceae had an average abundance of <0.5% in all conditions). Red circles are placed before the genera of periodontitis-associated “red-complex” bacteria. unclass. = unclassified (only shown at family-level); *adjusted p≤0.05 and **adjusted p<0.01 between the control and nitrate conditions, according to an ANCOMII analysis (for the complete dataset, see Supplementary Spreadsheet).

Supplementary Table 2A: OTU classification of genera with significant differences between the nitrate and control conditions

		5h						9h					
		Control			Nitrate			Control			Nitrate		
Genera	Species	X/12	Mean	SD	X/12	M	SD	X/12	Mean	SD	X/12	Mean	SD
Neisseria	NA	11	34.77	8.75	10	34.61	9.04	10	42.31	9.08	10	40.61	9.07
	flavescens	10	25.45	10.00	12	25.85	10.04	11	30.34	11.43	12	26.46	9.38
	subflava	8	20.99	8.91	8	21.91	8.76	8	18.55	7.80	8	18.68	7.39
	bacilliformis	10	11.56	8.20	9	8.75	7.03	5	1.89	1.29	10	5.58	3.78
	elongata	9	4.20	2.41	8	5.32	3.56	8	3.00	1.14	9	4.95	2.72
	subflava| pharyngis	9	1.88	0.56	8	2.20	0.69	7	1.94	0.75	7	2.18	0.77
	pharyngis	5	0.97	0.53	7	0.39	0.18	4	1.35	0.66	6	0.94	0.51
	Low abun. spp.	2	0.19	0.16	5	0.97	0.61	3	0.61	0.35	4	0.59	0.33
Rothia	*aeria| dentocariosa*	11	85.06	8.09	12	94.99	1.40	11	88.21	8.30	11	88.75	8.19
	mucilaginosa	8	6.20	2.52	8	4.13	1.30	2	2.82	2.15	3	1.47	1.00
	aeria	3	0.38	0.23	4	0.77	0.38	1	0.64	0.64	2	1.30	1.19
	Low abun. spp.	1	0.03	0.03	1	0.12	0.12	0	0.00	0.00	1	0.15	0.15
Streptococcus	NA	12	55.71	2.58	12	52.99	3.61	12	52.30	3.39	12	54.40	2.66
	anginosus| intermedius	10	8.85	3.27	10	9.93	4.12	10	12.64	4.63	10	10.69	3.64
	vestibularis| salivarius	12	8.92	2.69	11	10.77	2.96	11	9.27	2.15	10	9.78	3.01
	parasanguinis_II	12	8.30	1.34	12	10.01	1.53	12	7.32	1.63	12	6.62	1.52
	peroris|lactarius	12	6.41	1.16	11	5.82	1.22	12	3.68	0.75	11	5.00	1.43
	sp._oral_taxon_066 | parasanguinis_I	12	4.70	1.26	9	2.48	0.90	12	6.66	0.65	12	5.61	1.12
	sanguinis	11	2.82	0.77	12	3.87	1.43	10	3.64	1.77	11	3.95	1.32
	Low abun. spp.	12	3.40	0.40	12	3.02	0.48	12	3.58	0.47	12	3.43	0.60
	cristatus	8	0.91	0.55	7	1.10	0.55	9	0.90	0.31	6	0.53	0.18
Veillonella	*rogosae|parvula*	12	34.08	7.95	12	30.20	7.05	12	39.47	6.75	12	31.71	6.05
	parvula| denticariosi	12	13.00	2.63	12	19.58	5.22	12	16.65	3.03	12	21.03	3.38
	atypica	12	21.01	6.65	11	15.40	4.97	10	15.74	4.70	11	14.98	4.68
	dispar|atypica	10	12.45	4.22	11	10.33	3.82	11	9.97	3.75	11	8.70	3.12
	NA	12	8.24	2.84	12	10.67	3.37	12	8.19	2.22	12	11.68	2.54
	parvula	12	3.53	0.65	12	3.67	0.56	12	3.55	0.64	12	3.96	0.54
	sp._oral_taxon_780	7	1.62	0.96	6	1.71	1.40	6	2.78	2.43	5	1.42	1.12
	rogosae	10	1.72	0.46	11	1.97	0.64	12	2.05	0.31	11	1.54	0.43
	sp._oral_taxon_917	5	1.22	1.14	1	2.35	2.35	3	0.04	0.02	3	2.10	2.08
	dispar	11	1.25	0.48	10	1.89	0.62	12	0.94	0.29	10	1.45	0.40
	dispar|parvula	12	1.54	0.63	10	1.65	0.60	10	0.51	0.17	11	0.80	0.21
	Low abun. spp.	7	0.34	0.28	3	0.57	0.52	7	0.13	0.04	7	0.64	0.45
Porphyromo-nas	NA	11	35.70	4.85	9	32.85	9.26	11	58.68	9.34	10	48.09	8.78
	endodontalis|sp._oral_taxon_285	12	42.95	7.04	10	49.83	10.17	10	21.97	8.44	9	28.82	9.02
	sp._oral_taxon_930	7	19.09	6.47	4	7.27	3.69	8	18.84	6.92	3	12.32	6.57
	sp._oral_taxon_275 |catoniae	3	1.65	0.94	0	0.00	0.00	1	0.51	0.51	3	1.87	1.66
	Low abun. spp.	2	0.62	0.57	1	0.33	0.33	0	0.00	0.00	1	0.56	0.56
	pasteri	0	0.00	0.00	1	1.39	1.39	0	0.00	0.00	0	0.00	0.00
Fusobacterium	NA	12	62.01	7.84	11	70.18	10.65	12	66.12	9.49	10	51.38	10.42
	periodonticum	8	21.27	7.06	5	10.53	5.68	7	21.74	7.96	8	18.68	5.24
	nucleatum_subsp._ polymorphum| nucleatum_subsp._ animalis	5	14.83	6.41	2	9.77	6.80	3	11.95	8.50	2	12.86	8.70
	nucleatum_subsp._ polymorphum| sp._oral_taxon_203	1	1.04	1.04	1	1.19	1.19	0	0.00	0.00	0	0.00	0.00
	Low abun. spp.	2	0.85	0.63	0	0.00	0.00	1	0.19	0.19	1	0.42	0.42

Low abundance spp. are species with <1% average abundance in each condition grouped together. NA is not classified using our criteria.

Supplementary Table 2B: continuation of OTU classification of genera with significant differences between the nitrate and control conditions

		5h						9h					
		Control			Nitrate			Control			Nitrate		
**Genera**	**Species**	**X/12**	**Mean**	*SD*	**X/12**	**M**	*SD*	**X/12**	**Mean**	*SD*	**X/12**	**Mean**	*SD*
Prevotella	***pallens***	**12**	**31.23**	*4.81*	**12**	**28.13**	*4.91*	**12**	**21.02**	*4.81*	**12**	**27.23**	*4.86*
	**NA**	**12**	**16.37**	*4.76*	**11**	**19.57**	*5.00*	**9**	**10.81**	*3.25*	**7**	**13.77**	*6.14*
	nigrescens	**11**	**11.25**	*3.18*	**12**	**19.48**	*5.89*	**11**	**13.39**	*3.14*	**9**	**10.70**	*3.71*
	oris	**12**	**7.68**	*2.34*	**10**	**7.33**	*2.03*	**10**	**8.92**	*2.88*	**10**	**10.16**	*4.68*
	intermedia	**10**	**7.74**	*3.42*	**7**	**5.80**	*2.49*	**8**	**6.81**	*3.49*	**5**	**4.12**	*2.37*
	scopos	**7**	**6.49**	*3.79*	**5**	**3.06**	*2.00*	**6**	**8.95**	*6.31*	**6**	**4.42**	*2.93*
	**Low abun. spp.**	**11**	**4.42**	*1.28*	**10**	**3.69**	*0.87*	**12**	**4.65**	*1.02*	**8**	**3.22**	*1.26*
	sp._oral_taxon_317| shahii	**6**	**1.58**	*0.98*	**3**	**1.92**	*1.50*	**5**	**8.72**	*4.49*	**6**	**2.83**	*1.19*
	sp._oral_taxon_306| sp_oral_taxon_314	**9**	**2.28**	*0.93*	**7**	**1.36**	*0.42*	**7**	**3.15**	*1.04*	**7**	**3.07**	*1.36*
	sp._oral_taxon_306	**9**	**2.51**	*0.84*	**7**	**2.68**	*1.40*	**7**	**0.94**	*0.32*	**5**	**2.15**	*1.29*
	sp._oral_taxon_472| loescheii	**5**	**0.52**	*0.39*	**6**	**0.65**	*0.30*	**6**	**2.09**	*1.39*	**7**	**4.87**	*2.77*
	sp._oral_taxon_942	**5**	**1.93**	*1.60*	**2**	**0.57**	*0.54*	**1**	**0.81**	*0.81*	**2**	**4.59**	*3.09*
	sp._oral_taxon_292| sp._oral_taxon_300	**7**	**0.64**	*0.26*	**5**	**0.94**	*0.57*	**7**	**3.94**	*1.65*	**4**	**0.88**	*0.75*
	sp._oral_taxon_443	**5**	**0.30**	*0.19*	**3**	**0.55**	*0.50*	**6**	**1.14**	*0.43*	**3**	**3.04**	*2.76*
	histicola| sp._oral_taxon_313	**8**	**0.79**	*0.32*	**5**	**1.57**	*1.15*	**5**	**1.21**	*0.69*	**4**	**0.43**	*0.31*
	pleuritidis	**4**	**0.84**	*0.65*	**3**	**0.62**	*0.49*	**3**	**0.83**	*0.70*	**4**	**1.29**	*0.80*
	histicola	**7**	**1.09**	*0.48*	**6**	**0.89**	*0.40*	**5**	**0.57**	*0.21*	**5**	**0.63**	*0.27*
	fusca	**2**	**1.02**	*0.70*	**2**	**0.11**	*0.09*	**2**	**0.77**	*0.53*	**2**	**0.89**	*0.63*
	sp._oral_taxon_313	**8**	**1.02**	*0.29*	**5**	**0.71**	*0.37*	**4**	**0.65**	*0.35*	**4**	**0.33**	*0.17*
	nanceiensis	**5**	**0.33**	*0.21*	**3**	**0.36**	*0.32*	**6**	**0.63**	*0.25*	**2**	**1.38**	*1.31*
Alloprevotella	***sp._oral_taxon_473***	**11**	**67.26**	*8.74*	**8**	**44.66**	*11.06*	**10**	**65.47**	*11.01*	**12**	**69.60**	*10.82*
	rava	**10**	**17.38**	*7.99*	**8**	**17.27**	*8.29*	**7**	**6.40**	*3.09*	**7**	**18.33**	*9.38*
	tannerae	**11**	**11.63**	*4.36*	**10**	**27.89**	*10.42*	**8**	**12.36**	*4.39*	**6**	**3.15**	*1.76*
	sp._oral_taxon_308| sp._oral_taxon_914	**6**	**2.47**	*1.37*	**3**	**4.09**	*2.95*	**5**	**7.22**	*4.11*	**5**	**4.18**	*2.90*
	sp._oral_taxon_308	**4**	**1.23**	*0.78*	**3**	**5.70**	*5.54*	**3**	**4.67**	*4.43*	**3**	**4.73**	*3.08*
	sp._oral_taxon_912| sp._oral_taxon_913	**1**	**0.02**	*0.02*	**1**	**0.38**	*0.38*	**4**	**3.87**	*2.83*	**0**	**0.00**	*0.00*
Leptotrichia	**NA**	**12**	**81.79**	*5.54*	**12**	**75.19**	*5.91*	**12**	**75.77**	*5.60*	**12**	**86.17**	*5.03*
	wadei| sp._oral_taxon_417	**7**	**5.20**	*3.29*	**6**	**5.76**	*2.57*	**6**	**8.11**	*3.91*	**5**	**4.07**	*1.80*
	hongkongensis	**6**	**3.21**	*1.49*	**4**	**8.00**	*3.66*	**6**	**3.14**	*1.15*	**4**	**2.41**	*1.20*
	sp._oral_taxon_212| sp._oral_taxon_215	**3**	**3.75**	*3.45*	**2**	**3.13**	*2.77*	**5**	**3.90**	*3.21*	**2**	**4.17**	*3.91*
	sp._oral_taxon_215	**4**	**2.33**	*1.94*	**3**	**2.72**	*1.93*	**8**	**4.60**	*1.92*	**1**	**0.98**	*0.98*
	sp._oral_taxon_498	**6**	**2.17**	*0.98*	**5**	**3.34**	*1.58*	**5**	**2.03**	*0.79*	**2**	**0.98**	*0.81*
	shahii| sp._oral_taxon_879	**5**	**1.43**	*0.77*	**3**	**1.27**	*0.79*	**3**	**1.11**	*0.62*	**1**	**0.83**	*0.83*
	**Low abundance spp.**	**2**	**0.13**	*0.09*	**2**	**0.58**	*0.40*	**5**	**1.34**	*0.76*	**2**	**0.38**	*0.26*
Oribacterium	***sinus|parvum***	**12**	**71.78**	*3.87*	**11**	**69.96**	*8.76*	**11**	**66.41**	*8.15*	**12**	**86.58**	*5.28*
	parvum	**10**	**14.52**	*2.99*	**9**	**16.42**	*8.13*	**10**	**10.24**	*2.53*	**7**	**9.02**	*3.81*
	**NA**	**9**	**7.19**	*2.31*	**7**	**4.62**	*1.47*	**10**	**8.78**	*2.77*	**2**	**1.21**	*0.99*
	sp._oral_taxon_078	**5**	**4.44**	*2.27*	**4**	**5.14**	*3.29*	**3**	**3.80**	*3.03*	**1**	**1.47**	*1.47*
	sinus	**6**	**1.91**	*0.82*	**6**	**3.43**	*1.65*	**4**	**2.43**	*1.43*	**2**	**1.72**	*1.56*
	**Low abundance spp.**	**2**	**0.16**	*0.12*	**3**	**0.44**	*0.27*	**0**	**0.00**	*0.00*	**0**	**0.00**	*0.00*

Low abundance spp. are species with <1% average abundance in each condition grouped together. NA is not classified using our criteria.

Supplementary Table 3: OTU classification of dominant and clinically relevant genera that did not change significantly on genera-level

		5h						9h					
		Control			Nitrate			Control			Nitrate		
**Genera**	**Species**	**X/12**	**Mean**	*SD*	**X/12**	**M**	*SD*	**X/12**	**Mean**	*SD*	**X/12**	**Mean**	*SD*
Haemophilus	***sputorum***	**12**	**42.36**	*9.24*	**9**	**48.71**	*11.41*	**11**	**44.30**	*8.20*	**11**	**49.13**	*10.84*
	**NA**	**12**	**28.17**	*7.89*	**11**	**16.80**	*7.39*	**11**	**18.09**	*5.47*	**11**	**16.91**	*6.93*
	pittmaniae	**10**	**17.16**	*8.21*	**10**	**15.07**	*8.61*	**10**	**19.32**	*7.51*	**10**	**17.01**	*8.97*
	sp._oral_taxon_036	**9**	**4.75**	*3.16*	**8**	**4.64**	*3.69*	**9**	**4.53**	*2.42*	**8**	**2.65**	*1.82*
	Parainfluenzae	**10**	**1.60**	*0.69*	**9**	**1.41**	*0.56*	**9**	**9.55**	*8.26*	**10**	**1.13**	*0.60*
	paraphrohaemolyticus| parahaemolyticus	**6**	**3.91**	*2.92*	**6**	**3.37**	*2.74*	**6**	**2.60**	*1.59*	**7**	**2.40**	*1.65*
	**Low abundance spp.**	**11**	**2.05**	*0.46*	**10**	**1.67**	*0.43*	**10**	**1.62**	*0.41*	**10**	**2.42**	*0.76*
Gemella	**NA**	**12**	**85.20**	*2.89*	**12**	**85.71**	*2.18*	**12**	**88.38**	*0.82*	**12**	**86.09**	*3.42*
	haemolysans	**12**	**6.53**	*0.63*	**11**	**6.50**	*0.73*	**12**	**5.78**	*0.36*	**12**	**5.96**	*0.53*
	haemolysans|sanguinis	**12**	**4.34**	*0.74*	**12**	**3.92**	*0.53*	**10**	**2.94**	*0.45*	**12**	**3.71**	*0.64*
	sanguinis	**11**	**2.68**	*1.53*	**12**	**3.14**	*1.77*	**10**	**1.38**	*0.41*	**9**	**1.30**	*0.68*
	morbillorum	**11**	**1.25**	*0.42*	**9**	**0.74**	*0.44*	**11**	**1.52**	*1.01*	**9**	**2.95**	*2.05*
Granulicat-ella	***adiacens***	**12**	**64.98**	*7.96*	**12**	**74.15**	*8.08*	**12**	**76.70**	*9.06*	**12**	**77.55**	*7.03*
	elegans	**10**	**35.02**	*7.96*	**8**	**25.85**	*8.08*	**8**	**23.30**	*9.06*	**8**	**22.45**	*7.03*
Aggregati-bacter	**NA**	**12**	**89.67**	*5.75*	**11**	**77.80**	*9.57*	**11**	**85.03**	*7.99*	**10**	**78.11**	*10.66*
	sp._oral_taxon_458	**6**	**9.27**	*5.88*	**6**	**3.71**	*2.20*	**6**	**2.73**	*1.16*	**5**	**0.94**	*0.43*
	sp._oral_taxon_512| segnis	**3**	**0.34**	*0.19*	**6**	**6.68**	*4.90*	**6**	**2.83**	*1.29*	**6**	**2.55**	*0.87*
	sp._oral_taxon_513| sp._oral_taxon_898	**1**	**0.19**	*0.19*	**0**	**0.00**	*0.00*	**0**	**0.00**	*0.00*	**2**	**8.36**	*8.33*
	aphrophilus| paraphrophilus	**2**	**0.46**	*0.44*	**3**	**2.64**	*1.78*	**3**	**0.14**	*0.08*	**2**	**0.54**	*0.45*
	**Low abundance spp.**	**2**	**0.08**	*0.07*	**1**	**0.83**	*0.83*	**3**	**0.93**	*0.83*	**3**	**1.17**	*1.03*
Treponema	**NA**	**9**	**55.07**	*11.75*	**7**	**33.35**	*11.20*	**3**	**17.01**	*9.52*	**5**	**26.88**	*10.45*
	sp._oral_taxon_257	**4**	**10.51**	*4.64*	**3**	**4.57**	*2.66*	**1**	**2.43**	*2.43*	**2**	**7.14**	*4.90*
	sp._oral_taxon_262	**3**	**1.56**	*0.93*	**3**	**3.86**	*2.79*	**2**	**3.82**	*2.88*	**2**	**4.17**	*2.99*
	sp._oral_taxon_249	**0**	**0.00**	*0.00*	**1**	**1.39**	*1.39*	**1**	**8.33**	*8.33*	**0**	**0.00**	*0.00*
	sp._oral_taxon_234| sp._oral_taxon_237	**3**	**2.63**	*1.68*	**3**	**5.68**	*3.18*	**0**	**0.00**	*0.00*	**0**	**0.00**	*0.00*
	sp._oral_taxon_246| sp._oral_taxon_247	**0**	**0.00**	*0.00*	**1**	**1.39**	*1.39*	**0**	**0.00**	*0.00*	**1**	**2.78**	*2.78*
	sp._oral_taxon_231	**0**	**0.00**	*0.00*	**0**	**0.00**	*0.00*	**1**	**1.39**	*1.39*	**1**	**1.39**	*1.39*
	sp._oral_taxon_508	**1**	**0.95**	*0.95*	**1**	**1.67**	*1.67*	**0**	**0.00**	*0.00*	**0**	**0.00**	*0.00*
	**Low abundance spp.**	**1**	**0.48**	*0.48*	**1**	**1.39**	*1.39*	**0**	**0.00**	*0.00*	**1**	**0.69**	*0.69*
	sp._oral_taxon_237	**1**	**1.04**	*1.04*	**1**	**1.39**	*1.39*	**0**	**0.00**	*0.00*	**0**	**0.00**	*0.00*
Tannerella	**NA**	**6**	**34.40**	*12.47*	**5**	**27.64**	*11.18*	**6**	**41.67**	*13.88*	**5**	**38.89**	*14.11*
	forsythia	**7**	**48.93**	*13.32*	**7**	**39.03**	*12.63*	**3**	**22.92**	*12.10*	**2**	**11.11**	*8.54*
	sp._oral_taxon_286	**0**	**0.00**	*0.00*	**0**	**0.00**	*0.00*	**1**	**2.08**	*2.08*	**0**	**0.00**	*0.00*

Low abundance spp. are species with <1% average abundance in each condition grouped together. NA is not classified using our criteria.

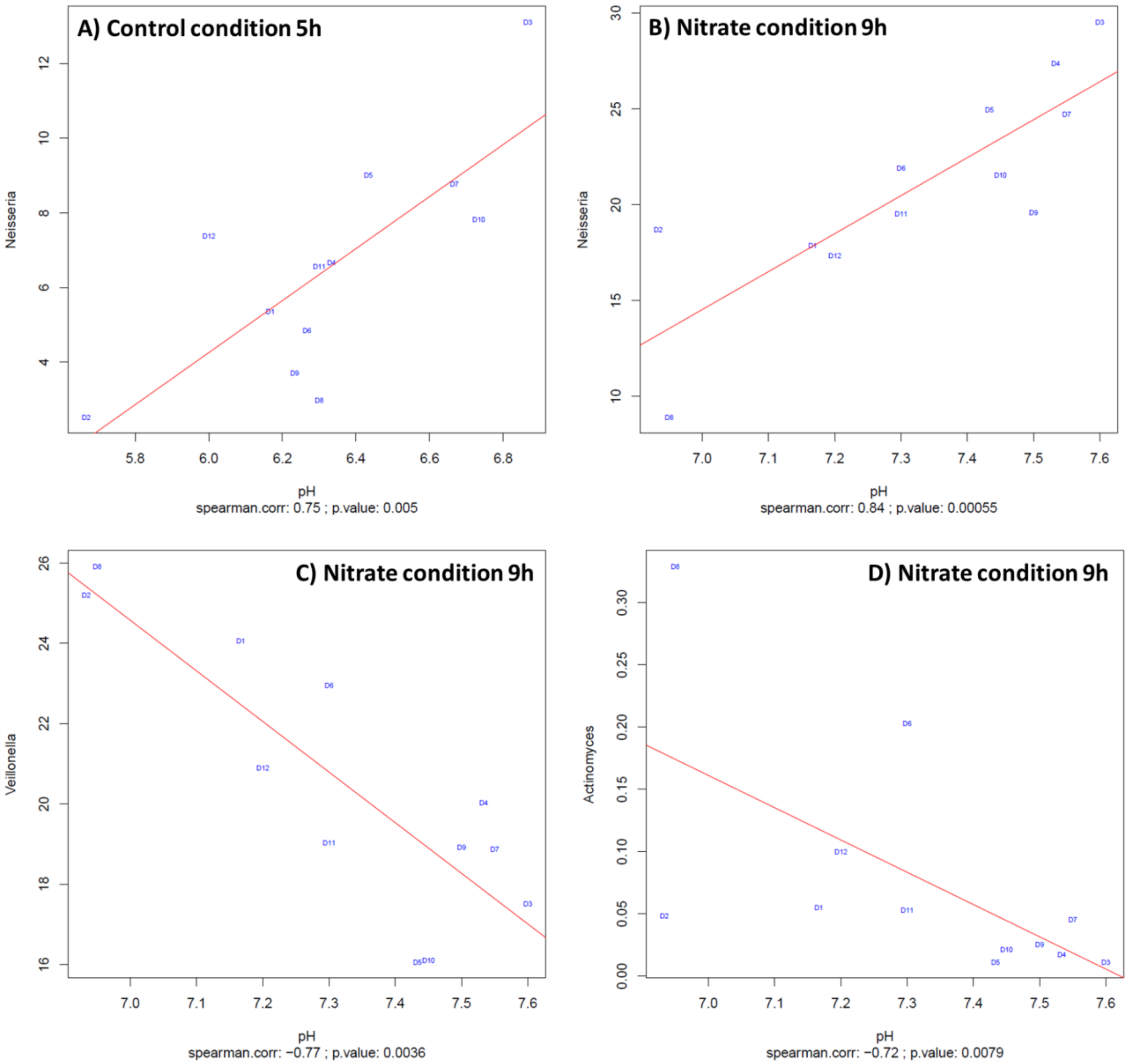

Supplementary Fig. 4: Significant correlations between genera and pH. All significant correlations (adjusted $p < 0.05$) between classified genera and pH are shown. A) Correlation between *Neisseria* and pH in the control condition at 5h. In the other sections, the correlations between *Neisseria* (B), *Veillonella* (C) and *Actinomyces* (D) are shown in the nitrate condition at 9h. In the graphs, unadjusted p-values are shown. The adjusted p-values for A, B, C and D were 0.025, 0.007, 0.016 and 0.017, respectively.
